# Novel Methylselenoesters as Antiproliferative Agents

**DOI:** 10.3390/molecules22081288

**Published:** 2017-08-02

**Authors:** Nuria Díaz-Argelich, Ignacio Encío, Daniel Plano, Aristi P. Fernandes, Juan Antonio Palop, Carmen Sanmartín

**Affiliations:** 1 Department of Organic and Pharmaceutical Chemistry, Faculty of Pharmacy and Nutrition, University of Navarra, Irunlarrea 1, E-31008 Pamplona, Spain; ndiaz@alumni.unav.es (N.D.-A.); dplano@unav.es (D.P.); jpalop@unav.es (J.A.P.); 2 Oncology and Hematology Section, IdiSNA, Navarra Institute for Health Research, Irunlarrea 3, E-31008 Pamplona, Spain; 3 Division of Biochemistry, Department of Medical Biochemistry and Biophysics (MBB), Karolinska Institutet, SE-171 77 Stockholm, Sweden; Aristi.Fernandes@ki.se; 4 Department of Health Sciences, Public University of Navarra, Avda. Barañain s/n, E-31008 Pamplona, Spain; ignacio.encio@unavarra.es

**Keywords:** methylselenoester, methylselenol release, cytotoxicity, cell cycle arrest, cell death, thioredoxin reductase

## Abstract

Selenium (Se) compounds are potential therapeutic agents in cancer. Importantly, the biological effects of Se compounds are exerted by their metabolites, with methylselenol (CH_3_SeH) being one of the key executors. In this study, we developed a new series of methylselenoesters with different scaffolds aiming to modulate the release of CH_3_SeH. The fifteen compounds follow Lipinski’s Rule of Five and with exception of compounds **1** and **14**, present better drug-likeness values than the positive control methylseleninic acid. The compounds were evaluated to determine their radical scavenging activity. Compound **11** reduced both DPPH and ABTS radicals. The cytotoxicity of the compounds was evaluated in a panel of five cancer cell lines (prostate, colon and lung carcinoma, mammary adenocarcinoma and chronic myelogenous leukemia) and two non-malignant (lung and mammary epithelial) cell lines. Ten compounds had GI_50_ values below 10 μM at 72 h in four cancer cell lines. Compounds **5** and **15** were chosen for further characterization of their mechanism of action in the mammary adenocarcinoma cell line due to their similarity with methylseleninic acid. Both compounds induced G_2_/M arrest whereas cell death was partially executed by caspases. The reduction and metabolism were also investigated, and both compounds were shown to be substrates for redox active enzyme thioredoxin reductase.

## 1. Introduction

Cancer is one of the leading causes of mortality and morbidity worldwide. Current chemotherapy is not completely satisfactory [1] and the development of new drugs is an urgent need. In the last few years, scientific evidence has backed the rationale for studying the mechanism of selenium-containing compounds as cancer therapeutic agents [2]. The role of Se is highly versatile due to multiple factors that determine its activity. It has been described both as a chemopreventive and cytotoxic agent [2,3,4] on account of the dual role as antioxidant and pro-oxidant, which is highly dependent on dose, chemical species and the redox state of the cell. Chemotherapeutic activity of Se compounds is mainly based in a multi-targeting effect which triggers complex cascades of death signaling and inhibits tumor formation and metastasis in animal models [2,5,6,7]. Because of this, the utility of Se compounds for the treatment of tumoral diseases and even drug-resistant cancers offers an interesting pursuit.

However, Se activity relies in multiple factors such as chemical form, dose and metabolism. Results from in vitro studies, animal experiments and clinical trials suggest that the biological activities of Se are dependent on the type and the nature of metabolites derived from the Se derivative [8,9]. Metabolites, and not original molecules, seem to be the ultimate executors of Se biological activity for some Se compounds. Thus, understanding the metabolic routes of Se compounds is highly necessary [10,11]. Se biochemistry encompasses a complex net of interrelated intermediates that converge in two main metabolites: methylselenol (CH_3_SeH) and hydrogen selenide (H_2_Se). Among all Se metabolites, it is largely accepted that CH_3_SeH stands out as a key executor for Se anticancer activity [3].

The volatile nature and high reactivity of CH_3_SeH obliges to the use of precursors with the ability to release it through hydrolysis, chemical reactions or cellular metabolism. Different approaches have been investigated. Methylselenocysteine requires the action of a ß-lyase to release the metabolite, but it has shown some promising results, especially in vivo [2,12,13]. The prodrug/enzyme system of selenomethionine/l-methioninase is considered to be less potent since they are quite inert and present lower redox activity [14]. Methylseleninic acid (MSA) is a synthetic molecule which has been broadly studied both in vivo and in vitro [15,16,17] and it is considered one of the best CH_3_SeH precursors [14].

In addition to these classical precursors, our group has been synthesizing during the last decade several methylseleno derivatives [18,19,20,21,22]. These compounds exhibited significant anticancer activity compared to other alkyl derivatives against a broad spectrum of cancer cells both in vitro and in vivo. Moreover, we demonstrated that *Se*-methylselenourea derivatives were able to release CH_3_SeH in aqueous systems [23]. Recently, we identified a novel series of selenoester derivatives in which the presence of a methylselenoester moiety substantially improved the antiproliferative activity of the corresponding selenoglycolic acids [24]. Continuing with this work, herein we propose an extended series of molecules bearing this functionality. The methylselenoester entity can be easily attacked by a nucleophile such as water, as one possible mechanism to deliver CH_3_SeH. The release of the key metabolite in aqueous medium could therefore be modulated through the chemical features of the core of the molecule.

**Figure 1:**
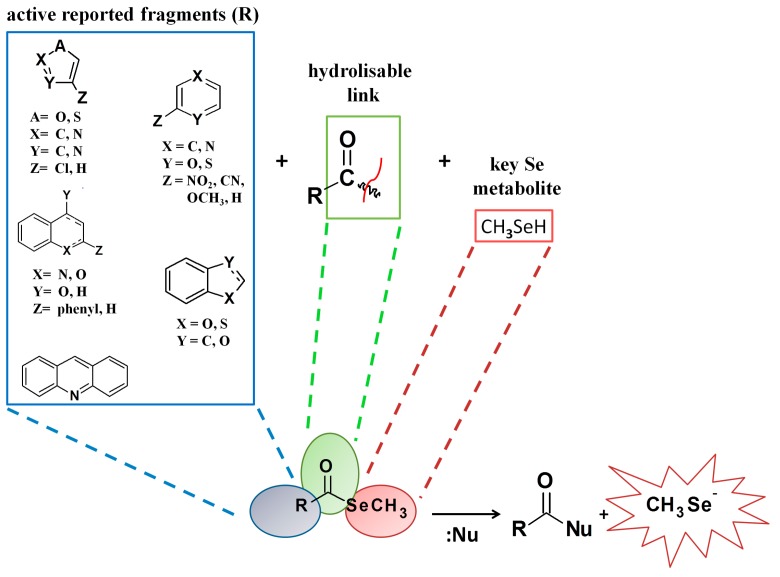
Fragment-based design of the novel methylselenoesters.

Using a fragment-based approach, different aromatic or heteroaromatic rings were selected to ensure enough chemical diversity to either hinder or facilitate the hydrolysis. In addition, we chose fragments that are present in compounds which have been reported active as anticancer agents by our group [19] and the literature: thienyl [25,26], isoxazolyl [27,28], furyl [29], chromonyl [30,31], pyrazinyl [32], pyridyl [33], thiazolyl [34], benzo[*b*]thienyl [35], quinolyl and phenylquinolyl [36,37,38] or acridinyl [39,40] as well as substituted aromatic rings (Figure 1).

Fifteen new compounds were synthesized and their ability to release CH_3_SeH in a modulated way was investigated. In addition, given the redox-modulating properties of Se compounds and based on previous antioxidant results of our work [24,41], the radical scavenging activity was also evaluated. All the compounds were screened against a panel of five cancer cell lines and two non-malignant cell lines. Moreover, two compounds were chosen to further perform mechanistic studies, including modulation of cell cycle, cell death evaluation and interaction with redox active enzymes.

## 2. Results and Discussion

### 2.1. Chemistry

Based on our previous research, we designed a new series of methylselenoesters. The selenoester moiety was chosen to facilitate a nucleophilic attack (i.e., water) resulting in the release of the key metabolite CH_3_SeH. Diverse active aromatic and heteroaromatic fragments were linked to the methylselenoester in order to provide different hydrolysis modulation, by hindering or facilitating the reaction. Bifunctionalized molecules bearing two methylselenoester groups were also synthesized to analyze if the cytotoxicity could be enhanced.

**Scheme 1:**
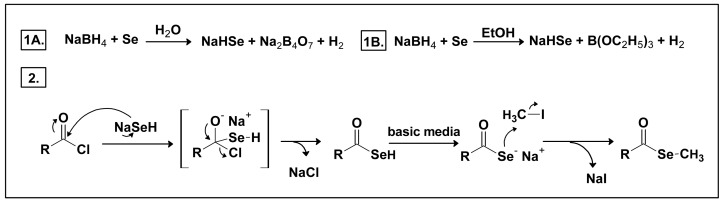
Synthesis of the methylselenoesters. Synthetic procedure and proposed reaction mechanism for the formation of the methylselenoesters.

A novel series of methylselenoesters was synthesized according to previously reported synthetic routes with few modifications [24,42,43]. The reaction of selenium powder and sodium borohydride in water (for compounds **4**, **6**, **8**, **9**, **11**, **14** and **15**) or ethanol (for compounds **1**–**3**, **5**, **7**, **10**, **12** and **13**) yielded sodium hydrogen selenide, which acts as a nucleophile. Substitution of the corresponding acyl chloride led to the sodium aroyl or heteroaroyl selenide, which was further methylated by an excess of methyl iodide (Scheme 1). Structures of the newly synthesized compounds are shown in Table 1.

Even though acyl chlorides are usually hydrolyzed in aqueous solvents, the compounds might be formed in water due to the fact that NaHSe is a superior nucleophile compared to water and reacts faster. Compounds **1**–**3**, **5**, **7**, **10**, **12** and **13**, however, could not be obtained in these conditions. Changing the solvent to absolute ethanol not only improved the solubility of the acyl chlorides, but enabled the formation of these compounds. For example, hydrolysis of extremely reactive acyl chlorides before reacting with NaHSe (i.e., the 4-nitrophenyl derivative) might be avoided in absolute ethanol. In addition, ethanol is less polar than water resulting in enhanced nucleophility of NaHSe, thus facilitating the nucleophilic substitution in more deactivated molecules, for instance in those with electron donors (i.e., the 4-methoxyphenyl derivative).

The purity of the newly synthesized compounds was assessed by thin layer chromatography (TLC) and elemental analysis. The compounds were purified mainly by recrystallization in different solvents or by column chromatography using methylene chloride as the eluent. Compounds **3**, **8**, **10** and **12** presented a troublesome purification with poor yields (<20%).

Structures were confirmed by infrared spectroscopy (IR), ^1^H-NMR, ^13^C-NMR and mass spectrometry (MS). Regarding IR, a characteristic strong peak corresponding to the carbonyl group appeared at 1620–1681 cm^−1^ whereas peaks corresponding to the methyl group (C–H_aliph_) appeared at 2918–2981 cm^−1^. In ^1^H-NMR, two microsatellites showing coupling of ^77^Se with ^1^H (*J*_Se–H_ = 5.2–5.6) were characteristic of the methyl group, whose chemical shift appeared at δ 2.28–2.64 ppm. MS data revealed that the base peak in almost all the molecules was the ion resulting from a 95-weight fragment loss, corresponding to CH_3_SeH. Interestingly, the base peak of the quinoline derivatives **9** and **10** had also lost the carbonyl group, even though compound **10** had an almost equal abundant ion (96%) which conserved it. For compound **13**, the two ions with and without the carbonyl group coexisted at almost the same abundance (100% and 92%). Compound **11** was the only molecule where the base peak did not correspond to the loss of CH_3_SeH but to a more fragmented ion. Compound **15** showed an interesting behavior. The base peak corresponded to the ion resulting from losing only one CH_3_SeH group. This did not happen with the other bifunctionalized molecule, compound **14**, whose more stable ion had lost both CH_3_SeH moieties in addition to the cyano group.

### 2.2. Methylselenol Release Studies

**Figure 2:**
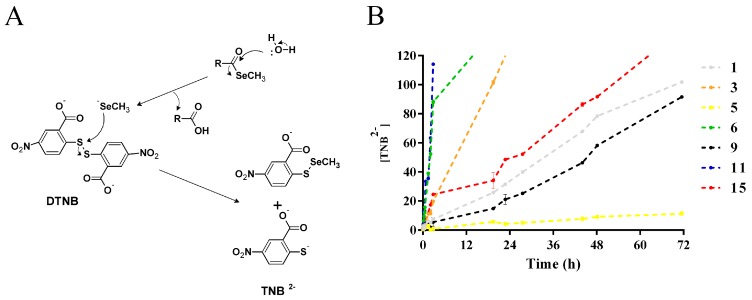
CH_3_SeH release rates are dependent on the chemical features of the molecule. Released CH_3_SeH was quantified through reaction with Ellman’s reagent (DTNB). (**A**) Proposed reaction between CH_3_SeH and DTNB; (**B**) Quantification of TNB^2−^ at different times. The compounds were tested at 100 μM in 200 μL of 100 mM phosphate buffer (1 mM EDTA, pH = 8). DTNB concentration was also 100 μM. Absorbance was followed at 412 nm over 72 h. A cysteine standard curve (7.5–150 μM) was used to calculate the concentration of TNB^2−^ over time. Compounds showing less than 15% hydrolysis at 72 h are not included, except for compound **5** as example. Error bars indicate SD of triplicates.

The new compounds were designed as CH_3_SeH precursors. The chemical features of the selenoester functional group facilitate a nucleophilic attack (i.e., with water) which would deliver the CH_3_SeH residue. In order to verify if CH_3_SeH could be released from the molecules by hydrolysis and if the rate differed among the compounds, the reaction of CH_3_SeH with 5,5′-dithiobis(2-nitrobenzoic acid) (DTNB or Ellman’s reagent) in aqueous environment was monitored [23,44]. Briefly, when the disulfide bridge of DTNB reacts with thiols or selenols, 2-nitro-5-thiobenzoate (TNB^2−^) is released and the mixed disulfide or selenylsulfide is formed. Each released CH_3_SeH produces one yellow TNB^2−^ species, whose absorbance can be quantified at 412 nm (Figure 2A). 

Results showed that the hydrolysis rate in these conditions varied among the compounds, as intended in the design of the molecules (Figure 2B). A fast release was observed for compounds **6** and **11**, which were completely hydrolyzed within the first two hours of incubation. CH_3_SeH was completely released from compound **3** at 24 h whereas a more sustained release corresponded, in this order, to compounds **15**, **1**, and **9**. However, the remaining compounds were hardly hydrolyzed (<15%) at 72 h. Unfortunately, compound **12** could not be tested due to reproducibility issues caused by lack of solubility in the assay conditions.

Results from this assay might not be predictive of the behavior of the compounds in other biological matrixes due to different testing conditions. In addition, other mechanisms rather than hydrolysis could lead to the release of methylselenol in the cell culture. However, we demonstrate that the chemical features are diverse enough to differentially modulate the lability of the carbonyl-selenium bond in this particular setting.

### 2.3. Theoretical Calculations of Molecular Properties

In order to provide a theoretical prediction of the drug-likeness properties of the new methylselenoesters, the freely accessible Molinspiration and Osiris DataWarrior programs were employed. 

#### 2.3.1. Molinspiration Calculations

Molinspiration calculations predict large values of logP, fitting all the compounds the recommended range of 0 ≤ logP ≤ 5 (Table 2). In fact, most of the derivatives present a logP value around the mean value of this range, which can be considered as the optimal relation between hydrophilicity and lypophilicity. Also, the theoretical values obtained are in accordance with the expected fact that structures with 3 aromatic rings (compounds **10** and **13**) should be the most lypophilic derivatives, showing higher logP values.

Furthermore, polar surface area (PSA) values equal to or greater than 140 are expected to exhibit poor intestinal absorption. All the values predicted are significatively below this threshold, pointing towards excellent intestinal absorption (Table 2).

To summarize, each of the fifteen selenoesters obtained shows good to excellent values for the calculated molecular descriptors and fulfills every facet of the Lipinski’s Rule of Five. This fact suggests that these CH_3_SeH precursors may show bioavailability, metabolic stability and transport properties comparable to known drugs.

#### 2.3.2. Osiris DataWarrior Calculations

Toxicity risks predicted by using Osiris-based calculations are shown in Table 3. Nine out of the fifteen compounds showed no toxicity for the four toxic parameters predicted. However, although the drug-likeness scores are low for all of the compounds, we hypothesized that this might be due to the presence of the Se atom, which is treated by the program as a hazardous element. Nevertheless, a wide range for this drug-likeness score can be found among the Se compounds synthesized. Noteworthy, thirteen compounds exhibit better drug-likeness values than the reference MSA. Compounds **12**, **4**, **7**, **15** and **5**, in this order, present the five highest values predicted.

### 2.4. Biological Studies

#### 2.4.1. Radical Scavenging Activity of the New Methylselenoesters

**Figure 3:**
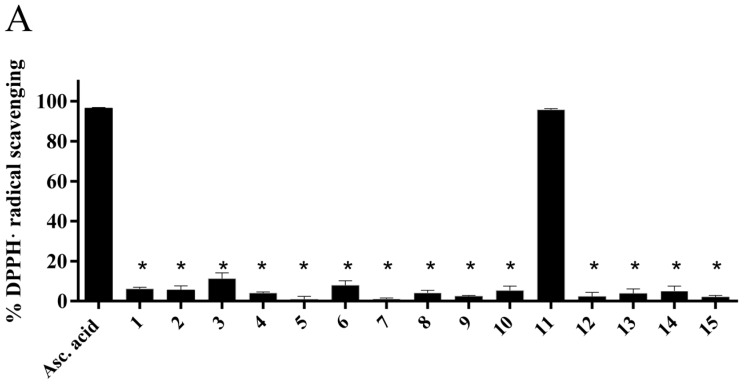

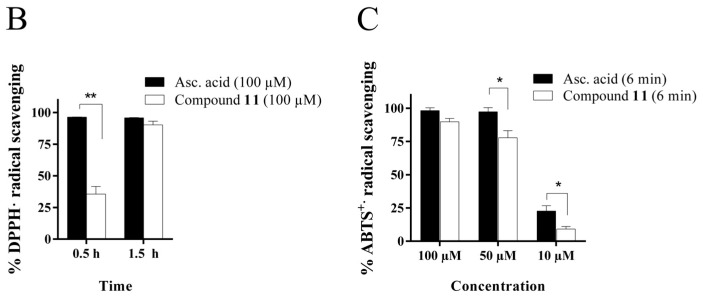
Compound **11** shows radical scavenging activity against DPPH^.^ and ABTS^+.^ radicals. (**A**) DPPH^.^ radical scavenging activity of the methylselenoesters and ascorbic acid (Asc. acid) (100 μM) after 3 h incubation. Results represent the mean ± SEM of at least three independent assays performed in triplicate; (**B**) DPPH^.^ radical scavenging activity of compound **11** at different times. Results represent the mean ± SEM of at least three independent assays performed in triplicate; (**C**) ABTS^+.^ radical scavenging activity of compound **11** at different concentrations after 6 min incubation. Results represent the mean ± SD of quadruplicates. * *p* < 0.05, ** *p* < 0.01 with respect to ascorbic acid.

Se compounds have a dual role as pro-oxidant and antioxidant, depending on the dose and the chemical species. In fact, the chemopreventive activity of Se is believed to be due to its antioxidant features through incorporation into selenoproteins. Aiming to have a first approach on the redox properties, we analyzed the radical scavenging capability of the novel methylselenoesters. First, the reduction of 1,1-diphenyl-2-picrylhydrazyl (DPPH) was measured. Compounds were tested at 100 μM up to 3 h and ascorbic acid was included as a positive control. Except for compound **11**, which showed similar activity to ascorbic acid after 1 h incubation, none of the compounds demonstrated radical scavenging properties (Figure 3A,B). To validate the antioxidant properties of compound **11**, the reduction of another radical, 2,2-azinobis(3-ethylbenzothiazoline-6-sulphonic acid) (ABTS^+**.**^), was also tested. The radical scavenging activity of compound **11** towards this radical was similar to ascorbic acid at 100 μM (Figure 3C) but at lower concentrations, ascorbic acid demonstrated greater antioxidant properties than compound **11**. 

#### 2.4.2. Cytotoxic Activity of the Novel Methylselenoesters 

The novel compounds were tested against a panel of different cancer lines: PC-3 (prostate adenocarcinoma), MCF7 (mammary adenocarcinoma), HTB-54 (lung carcinoma), K-562 (chronic myelogenous leukemia) and HT-29 (colorectal adenocarcinoma). Two non-malignant cell lines, BEAS-2B (bronchial epithelial) and 184B5 (mammary epithelial) were also included to evaluate the toxicity of the novel synthesized compounds. The antiproliferative activity was measured with the [3-(4,5-dimethylthiazol-2-yl)-2,5-diphenyltetrazolium bromide] (MTT) assay after 72 h treatment. Six concentrations ranging from 0.1 to 100 μM were tested. MSA is largely known as the main CH_3_SeH putative precursor and was therefore included as positive control. The following parameters were calculated: GI_50_ (concentration which reduces the growth by 50%), TGI (concentration completely inhibiting growth) and LC_50_ (concentration that kills 50% of the cells). Results are summarized in Table 4.

As shown in the table, every compound was able to inhibit proliferation, with compounds **2**–**6**, **8**–**11** and **14** presenting GI_50_ values below 10 μM in PC-3, MCF7, HT-29 and HTB-54, and greater than 10 μM in K-562, which was the most resistant cell line. GI_50_ values greater than 10 μM were also found for compounds **7**, **12** and **13** towards the prostate cancer cell line, and for compounds **1**, **7** and **15** against the lung cancer cell line, indicating that a higher dose of these compounds was needed to inhibit proliferation. When comparing compounds **4** and **5**, we found that the inclusion of an electron-withdrawing atom in the thiophene ring (compound 5) improved the activity in MCF7 (LC_50_ = 89.6 μM, LC_50_ = 35.9 μM, respectively) and HT-29 (LC_50_ = 47.5 μM, LC_50_ = 8.9 μM, respectively) but not in PC-3 or HTB-54. However, the presence of two methylseleno moieties did not particularly enhance potency, as neither compound **14** nor **15** had significantly lower GI_50_, TGI or LC_50_ values than the monofunctionalized molecules. Regarding TGI, higher values were generally found in HTB-54 and PC-3 cell lines than in MCF7 and HT-29. On the contrary, low TGI values (<10 μM) were found for compounds **5** and **14** in HTB-54 and for compounds **6**, **10**, and **15** in PC-3. Finally, when considering the LC_50_ parameter, all the compounds exhibited similar moderate potencies. In fact, the compounds were more cytostatic than cytotoxic due to high LC_50_ values (>30 μM).

The similar behavior of the compounds might support a shared mechanism of action for all of them. This led us to consider CH_3_SeH as the ultimate effector of the biological activity. However, we could not establish a clear correlation between CH_3_SeH release rates due to hydrolysis in the Ellmans’s assay and biological activity at the tested time period. The different conditions of both experiments could be an explanation for this fact. Besides, cell culture could modify the release rates: in addition to hydrolysis, other factors might be taking place, such as cell uptake before the molecules are hydrolyzed; cell metabolism leading to release of methylselenol; or concomitant reactions of the molecules with medium components. In fact, to evaluate if the acidic residue contributed to the activity in case of hydrolysis in the cell culture, we tested the corresponding acids. Given that CH_3_SeH has been largely characterized in mammary carcinoma [45,46], we chose this cell line (MCF7) for further studies. Although the residues had been selected according to an active fragment-based approach, the acidic forms were not toxic at 72 h (GI_50_ > 100 μM), thus supporting CH_3_SeH as the effector of the toxic activity.

Regarding the non-malignant cell lines, the positive control MSA was found to be toxic. Consistently, the new CH_3_SeH precursors were also toxic for BEAS-2B and 184B5. Their toxicity, however, was in the same range as MSA, which has been broadly studied both in vitro and in vivo [7,47].

Taking into account all the data, and on the basis of similarity to MSA kinetic parameters in both tumor MCF7 and non-malignant 184B5 cell lines, compounds **5** and **15** exhibited the most similar profile. In addition, compounds **5** and **15** did not violate Lipinski’s Rule of Five, presented good drug-likeness parameters and were among the five best compounds according to Osiris DataWarrior predictions. For these reasons, compounds **5** and **15** were selected for further characterization of their mechanism of action on the MCF7 cell line.

#### 2.4.3. Compounds **5** and **15** Lead to Cell Cycle Arrest in G_2_/M Phase

Cell cycle arrest is the target of many anticancer drugs, as it is the first step to decrease cell proliferation. To gain a better understanding of the mechanism of these compounds, the effect of compounds **5** and **15** on cell cycle distribution was analyzed. MCF7 cells were treated with increasing concentrations of both compounds for 24 h or with 15 μM **5** or 30 μM **15** for different times. The negative control was treated with vehicle, and camptothecin (6 μM) was used as a positive control. Samples were stained with propidium iodide using the Apo-Direct kit (BD Pharmingen, Madrid, Spain) and processed by flow cytometry. 

**Figure 4:**
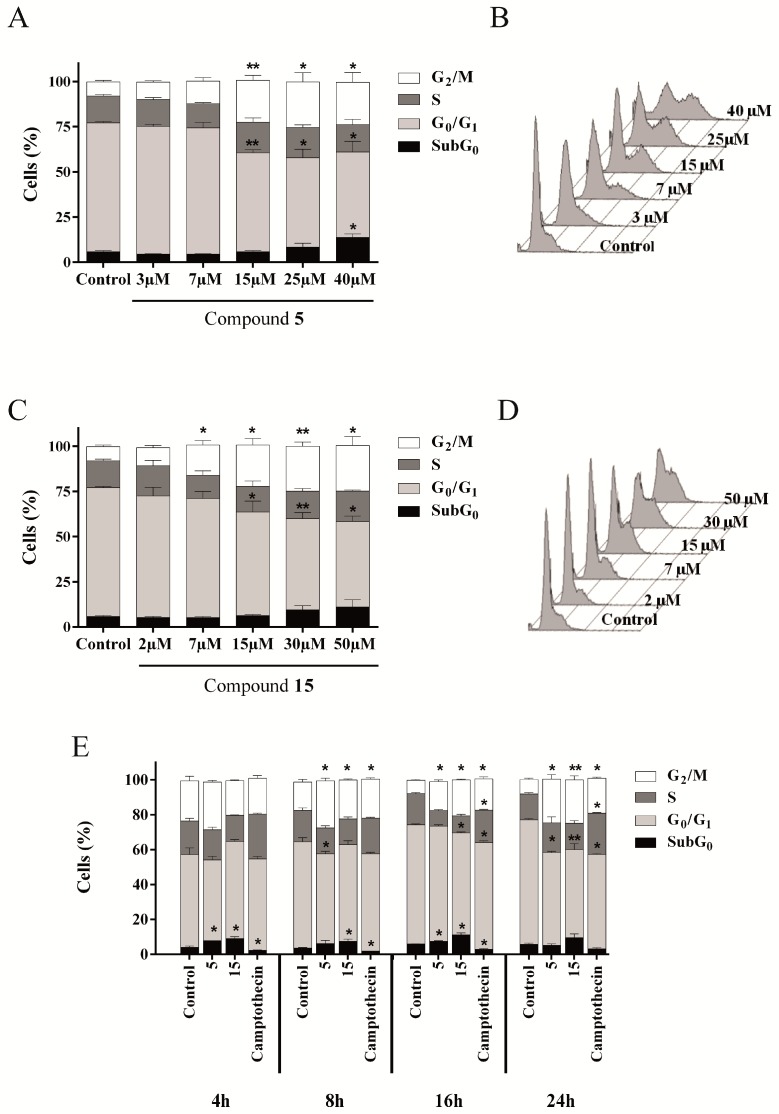
Compounds **5** and **15** induce cell cycle arrest in G_2_/M phase in MCF7. MCF7 cells were treated with different concentrations of compound **5**, **15** or vehicle (control) for 24 h or for different time periods. Cells were stained with propidium iodide with the Apo-Direct kit and analyzed by flow cytometry. (**A**) Quantification of cell cycle distribution after 24 h treatment with increasing concentrations of compound **5**; (**B**) Representative experiment of A; (**C**) Quantification of cell cycle distribution after 24 h treatment with increasing concentrations of compound **15**; (**D**) Representative experiment of C; (**E**) Cell cycle distribution after treatment with vehicle, 15 μM of compound **5** or 30 μM of compound **15** for different times. Results represent the mean ± SEM of at least three independent experiments performed in duplicate. * *p* < 0.05, ** *p* < 0.01.

As shown in Figure 4, treatment with compounds **5** and **15** increased the percentage of cells in G_2_/M phase. This increase was dose-dependent at 24 h and evident for compound **15** when cells were treated with 7 μM or higher concentrations whereas it was observed for compound **5** only from 15 μM treatment. In the time-course analysis, we found an increase in G_2_/M phase after 8 h treatment for both compounds that was correlated with a significant decrease in the G_0_/G_1_ phase at 8 h for compound **5** and 16 h for compound **15**. We conclude that these results indicate a cell cycle blockage in G_2_/M phase.

#### 2.4.4. Evaluation of Cell Death Mechanism Induced by Compounds **5** and **15**

Induction of cell death is the major aim of antitumor drugs. We analyzed cell death progression using the TUNEL technique (in the Apo-Direct kit), which is based on DNA fragmentation. MCF7 cells were treated with increasing concentrations of compounds **5** and **15** for 24 h or with 30 μM of compound **15** or 15 μM of compound **5** for different times. The negative control was treated with vehicle, and camptothecin (6 μM) was included as a positive control. 

**Figure 5:**
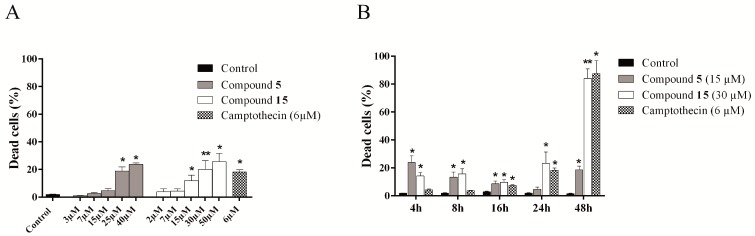
Cell death induction is dose and time-dependent for compound **15** in MCF7. MCF7 cells were treated with vehicle (control) or increasing doses of compound **5** or **15** for 24 h. For the time-course experiment, MCF7 cells were treated with vehicle (control), compound **5** (15 μM) or compound **15** (30 μM) for different times. Camptothecin (6 μM) was included as positive control. Cells were stained with the Apo-Direct kit (TUNEL assay) and analyzed by flow cytometry. (**A**) Dose-dependent cell death at 24 h; (**B**) Time-course analysis of cell death. Results show the mean ± SEM of at least three independent experiments performed in duplicate. * *p* < 0.05, ** *p* < 0.01.

Both compounds exhibited a dose-dependent effect at 24 h (Figure 5A), but only compound **15** caused cell death in a time-dependent manner (Figure 5B). Cell death was evident from only 4 h treatment for both compounds. However, whereas cell death induced by compound **15** rose from 20% of dead cells at 4 h up to 80% at 48 h, the highest values of cell death for compound **5** were observed after 4 h treatment.

Se compounds induce different types of cell death, among which apoptosis and autophagy are the most common. To further elucidate the pathway through which compounds **5** and **15** trigger cell death, we used Z-VAD-FMK, a pan-caspase inhibitor and wortmannin, a PI3K inhibitor which blocks autophagy. MCF7 cells were pre-incubated for 1 h with 50 μM of Z-VAD-FMK or 100 nM wortmannin and co-incubated with compound **5** (25 μM), compound **15** (30 μM) or vehicle (control) for 48 h. Treated cells without inhibitors were also included.

**Figure 6:**
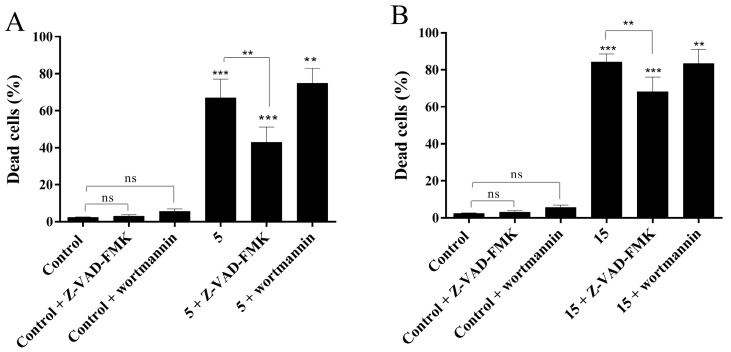
Cell death induced by compounds **5** and **15** involves the caspases pathway. MCF7 cells were pre-incubated with 50 μM of Z-VAD-FMK or 100 nM wortmannin for 1 h before treating cells with compound **5** (25 μM), compound **15** (30 μM) or vehicle (control) for 48 h. Cells were stained with the Apo-Direct kit (TUNEL assay) and analyzed by flow cytometry. (**A**) Cell death induced by compound **5** in the presence and absence of the inhibitors; (**B**) Cell death induced by compound **15** in the presence and absence of the inhibitors. Results show the mean ± SEM of at least three independent experiments performed in duplicate, ** *p* < 0.01, *** *p* < 0.001.

As shown in Figure 6, cell death induced by the novel methylselenoesters was not altered in the presence of wortmannin, ruling out autophagy as a mechanism of action. On the other hand, cell death was partially prevented when cells were treated in the presence of the pan-caspase inhibitor. Cell death induced by compound **5** was decreased by 36%, whereas the total number of dead cells was decreased by 14% in case of compound **15**. These results indicate that caspases are at least partially implicated in the cell death mechanism of these compounds. In fact, CH_3_SeH has been described to induce caspase-mediated cell death [47,48].

#### 2.4.5. Compounds **5** and **15** are Substrates for Thioredoxin Reductase But not for the Glutathione- Glutaredoxin System

Metabolism is crucial for Se compounds, as the biological activity is mainly exerted through their metabolites. It has been described that Se compounds can be metabolized by redox active enzymes [49,50]. Thus, in an attempt to refine possible metabolic pathways, we examined whether compounds **5** and **15** interacted with thioredoxin reductase (TrxR) and/or the gluthatione-glutaredoxin (GSH-Grx) system. We used MSA as control, as it is known to be reduced by both GSH and TrxR [51,52].

**Figure 7:**
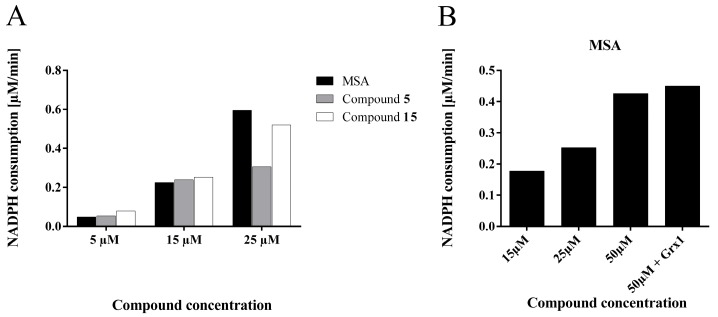
Compounds **5** and **15** are substrates for thioredoxin reductase but not for the glutathione-glutaredoxin system. (**A**) NADPH consumption indicating reduction of compounds **5**, **15** or MSA as control by thioredoxin reductase. The reaction mixture contained 100 nM TrxR1, 227 μM NADPH and the corresponding amount of compound in TE buffer (20 mM Tris, 2 mM EDTA pH = 8); (**B**) NADPH consumption indicating reduction of MSA by glutathione in the presence or absence of glutaredoxin. The reaction mixture contained the corresponding amount of compound, 0.1 M Tris, 2 mM EDTA pH = 8, 0.1 mg/mL BSA, 1 mM GSH, 200 μM NADPH, 0.008 OD/mL yeast GR and 1 μM hGrx1 when required. Only results for MSA are shown, as compounds **5** and **15** were not reduced.

Our results indicated that, while MSA was consistently reduced by both TrxR1 and GSH in accordance with previous studies, compounds **5** and **15** were substrates only for TrxR1 (Figure 7). The reduction by TrxR1 is considerably more efficient compared the spontaneous hydrolysis, indicating that this would facilitate the release of the active CH_3_SeH metabolite intracellularly.

## 3. Material and Methods

### 3.1. General Information

Melting points (m.p.) were determined with a FP82 + FP80 apparatus (Mettler, Greifensee, Switzerland). The NMR spectra (^1^H/400 MHz and ^13^C/100 MHz, Supplementary Materials) were recorded on a Ultrashield^TM^ 400 spectrometer (Bruker, Rheinstetten, Germany). The samples were dissolved in DMSO-*d*_6_ or CDCl_3_ and TMS was used as internal standard. IR spectra were obtained on a FT-IR Nexus spectrophotometer (Thermo Nicolet, Madison, WI, USA) using KBr pellets for solids or NaCl plates for oil compounds. Elemental analysis was performed on a CHN-900 Elemental Analyzer (LECO, Saint Joseph, MI, USA). HRMS were recorded using an Accurate-Mass TOF LC/MS 6220 (Agilent Technologies, Santa Clara, CA, USA). Only data from compounds **3**, **9**, **10**, **12** and **13** could be obtained due to poor volatilization of the remaining compounds. For TLC assays, Alugram SIL G7UV254 sheets (Macherey-Nagel; Düren, Germany) were used. Column chromatography was performed with silica gel 60 (Merck, Darmstadt, Germany). Chemicals were purchased from E. Merck, Panreac Química S.A. (Montcada i Reixac, Barcelona, Spain), Sigma-Aldrich Quimica, S.A. (Madrid, Spain) and Acros Organics (Janssen Pharmaceuticalaan, Geel, Belgium).

### 3.2. Chemistry

#### 3.2.1. General Procedure

Following a described procedure [24,42,43] with a few modifications, sodium borohydride was slowly added to a suspension of selenium powder in water at room temperature or in ethanol, N_2_ atmosphere and 0 °C, and stirred until the formation of the typical colorless solution of NaHSe. Then, the corresponding aroyl or heteroaroyl chloride was added. Temperature and time of reaction varied depending on the compounds. Methylation was achieved through the addition of methyl iodide (in excess). Purification was performed by several washings, recrystallization in different solvents or column chromatography. In those cases where the acyl chloride was not available, it was formed by the reaction of the corresponding carboxylic acid with SOCl_2_ for 1–8 h at reflux. Solvent was removed under vacuum by rotatory evaporation, and the product was then washed three times with dry toluene, which was also eliminated by rotatory evaporation.

#### 3.2.2. General Procedure for Compounds **4**, **6**, **8**, **9**, **11**, **14** and **15**

Sodium borohydride (4.15 mmol) was added to a suspension of powdered selenium (2 mmol) in water at room temperature. Discoloration to a colorless solution indicated the formation of sodium hydrogen selenide. Then, a solution of the acyl or heteroaroyl chloride in chloroform was added (2 mmol) and the mixture was stirred at 50 °C for 1 h, unless stated otherwise. For the bifunctionalized molecules (compounds **14** and **15**), 8.3 mmol of sodium borohydride, 4 mmol of powdered selenium and 2 mmol of the acyl chloride were used. Reaction was followed by TLC or IR. After filtering insoluble salts, an excess of methyl iodide (1.5 mL) was added and the reaction was heated at the same temperature (1 h, unless stated otherwise) until precipitation of the product or discoloration of the aqueous phase. The solid was filtered or extracted with methylene chloride, washed with slightly basic water and dried over Na_2_SO_4_. The solvent was eliminated under rotatory evaporation. Compounds were purified through recrystallization in appropriate solvents or column chromatography using methylene chloride as eluent (mobile phase).

*Methyl 2-thiophencarboselenoate* (**4**). From 2-thiophencarbonyl chloride. A yellow oil was obtained which was further purified by column chromatography. Yield: 26%. ^1^H-NMR (CDCl_3_): δ 2.42 (s, 3H, –SeCH_3_), 7.15 (dd, 1H, H_4_, *J*_4–3_ = 3.9, *J*_4–5_ = 4.9 Hz), 7.68 (dd, 1H, H_5_, *J*_5–3_ = 1.2 Hz, *J*_5–4_ = 4.9 Hz), 7.82 ppm (dd, 1H, H_3_, *J*_3–4_ = 3.9, *J*_3–5_ = 1.2 Hz). ^13^C-NMR (CDCl_3_): δ 5.7 (–SeCH_3_), 128.3 (C_4_), 131.8 (C_5_), 133.3 (C_3_), 144.3 (C_2_), 185.5 ppm (–C=O). IR (KBr): ν 3102 (w, C–H_arom_), 2932 (C–H_aliph_), 1662 cm^−1^ (–C=O). MS [*m*/*z* (% abundance)]: 206 (M^+•^ +1, 6), 111 (100), 83 (11). Elemental analysis calculated for C_6_H_6_OSSe (%): C: 35.13, H: 2.95; found: C: 35.20, H: 2.77.

*Methyl 5-isoxazolecarboselenoate* (**6**). From 5-isoxazolecarbonyl chloride. A brownish solid was obtained. Yield: 49%; m.p.: 41–42 °C. ^1^H-NMR (DMSO-*d_6_*): δ 2.42 (s, 3H, –SeCH_3_), 7.40 (d, 1H, H_4_, *J*_4–3_ = 2.0 Hz), 8.92 ppm (d, 1H, H_3_, *J*_3–4_ = 2.0 Hz). ^13^C-NMR (DMSO-*d_6_*): δ 6.0 (–SeCH_3_), 107.0 (C_4_), 153.3 (C_5_), 165.8 (C_3_), 183.2 ppm (–C=O). IR (KBr): ν 3158–3101 (w, C–H_arom_), 2931 (w, C–H_aliph_), 1693 (s, –CN), 1649 cm^−1^ (s, –C=O). MS [*m*/*z* (% abundance)]: 191 (M^+•^ +1, 15), 96 (100), 68 (63) 113 (46), 40 (57). Elemental analysis calculated for C_5_H_5_NO_2_Se (%): C: 31.60, H: 2.65, N: 7.37; found: C: 31.45, H: 3.03, N: 7.27.

*Methyl 1,3-benzodioxole-5-carboselenoate* (**8**). From 1,3-benzodioxole-5-carboxylic acid. A yellow solid was obtained, which was purified through recrystallization from diethyl ether:hexane (1:1). Yield: 15%; m.p.: 48–49 °C. ^1^H-NMR (CDCl_3_): δ 2.36 (s, 3H, –SeCH_3_), 6.05 (s, 2H, –CH_2_–), 6.84 (d, 1H, H_7_, *J*_7–6_ = 8.2 Hz), 7.35 (s, 1H, H_4_), 7.55 ppm (d, 1H, H_6_, *J*_6–7_ = 8.2 Hz). ^13^C-NMR (CDCl_3_): δ 4.9 (–SeCH_3_), 101.7 (C_2_), 106.5 (C_7_), 107.8 (C_4_), 123.2 (C_6_), 133.2 (C_5_), 147.8 (C_3a_), 151.8 (C_7a_), 192.3 ppm (–C=O). IR (KBr): ν 2918 (w, C–H_aliph_), 1655 (s, –C=O), 1036 (s, C–O–C sym) 925 cm^−1^ (s, C–O–C as). MS [*m*/*z* (% abundance)]: 149 (100), 121 (35), 65 (28), 91 (10). Elemental analysis calculated for C_9_H_8_O_3_Se (%): C: 44.46, H: 3.32; found: C: 44.17, H: 3.91.

*Methyl 3-quinolinecarboselenoate* (**9**). From 3-quinolinecarboxylic acid. Conditions: 15 min reaction with methyl iodide. A brown solid was obtained. Yield: 43%; m.p.: 74–75 °C. ^1^H-NMR (CDCl_3_): δ 2.48 (s, 3H, –SeCH_3_), 7.66 (dd, 1H, H_6_, *J*_6__–5_ = *J*_6–7_ = 7.5 Hz), 7.87 (dd, 1H, H_7_, *J*_7–6_ = 7.5 Hz, *J*_7–8_ = 7.7 Hz), 7.99 (d, 1H, H_5_, *J*_5–6_ = 7.5 Hz), 8.21 (d, 1H, H_8_, *J*_8–7_ = 7.7 Hz), 8.72 (s, 1H, H_4_), 9.35 ppm (s, 1H, H_2_). ^13^C-NMR (CDCl_3_): δ 6.0 (–SeCH_3_), 127.4 (C_6_), 128.5 (C_4a_), 129.5 (C_3_), 129.8 (C_5_), 132.0 (C_8_), 132.9 (C_7_), 136.9 (C_4_), 147.5 (C_2_), 149.9 (C_8a_), 193.5 ppm (–C=O). IR (KBr): ν 3027 (w, C–H_arom_), 2955 (w, C–H_aliph_), 1671 (s, –C=O), 1616 cm^−1^ (s, –C=N). MS [*m*/*z* (% abundance)]: 156 (88), 128 (100), 101 (55), 75 (35). Elemental analysis calculated for C_11_H_9_NOSe (%): C: 52.81, H: 3.63, N: 5.60; found: C: 53.01, H: 3.93, N: 5.39. HRMS calculated for C_11_H_10_NOSe^+^ [M + H]^+^: 251.9922; found: 251.9978.

*Methyl 6-bromochromone-2-carboselenoate* (**11**). From 6-bromochromone-2-carboxylic acid. The solid was purified through column chromatography. A yellow solid was obtained. Yield: 27%; m.p.: 156–157 °C. ^1^H-NMR (CDCl_3_): δ 2.46 (s, 3H, –SeCH_3_), 6.93 (s, 1H, H_3_), 7.52 (d, 1H, H_8_, *J*_8–7_ = 8.9), 7.86 (dd, 1H, H_7_, *J*_7–5_ = 2.4, *J*_7–8_ = 8.9), 8.33–8.35 ppm (m, 1H, H_5_). ^13^C-NMR (CDCl_3_): δ 5.7 (–SeCH_3_), 101.0 (C_3_), 120.2 (C_6_), 120.9 (C_8_), 126.3 (C_4a_), 129.0 (C_5_), 138.2 (C_7_), 154.6 (C_8a_), 157.1 (C_2_), 177.2 (C_4_), 190.6 ppm (–C=O). IR (KBr): ν 3039 (w, C–H_arom_), 2928 (w, C–H_aliph_), 1676 (s, –C=O), 1645 cm^−1^ (s, –COSe). MS [*m*/*z* (% abundance)]: 346 (M^+•^, 30), 251 (69), 282 (24), 223 (36), 169 (100), 88 (41), 69 (57). Elemental analysis calculated (%) for C_11_H_7_BrO_3_Se: C: 38.18, H: 2.04; found: C: 37.87, H: 2.21.

*Dimethyl 5-cyano-1,3-benzenedicarboselenoate* (**14**). From 5-cyano-1,3-benzenedicarboxylic acid. Conditions: 1.5 h reaction with NaHSe and 4 h reaction with methyl iodide. The solid was purified through column chromatography. A pink solid was obtained. Yield: 26%; m.p.: 167–168 °C. ^1^H-NMR (CDCl_3_): δ 2.51 (s, 6H, –SeCH_3_), 8.36 (d, 2H, H_4_ + H_6_, *J*_4–2_ = *J*_6–2_ = 2.7), 8.57 ppm (d, 1H, H_2_, *J*_2–4_ = *J*_2–6_ = 2.7). ^13^C-NMR (CDCl_3_): δ 6.6 (–SeCH_3_), 114.7 (C_5_), 117.1 (–CN), 129.1 (C_4_), 134.5 (C_2_), 141.0 (C_1_ + C_3_), 192.9 ppm (–C=O). IR (KBr): ν 3073 (w, C–H_arom_), 2935 (w, C–H_aliph_), 2229 (s, –CN), 1670 cm^−1^ (s, –C=O). MS [*m*/*z* (% abundance)]: 252 (59), 129 (100), 101 (84). Elemental analysis calculated for C_11_H_9_O_2_Se_2_N (%): C: 38.26, H: 2.61, N: 4.05; found: C: 38.36, H: 3.04, N: 3.98.

*Dimethyl 2,5-furandicarboselenoate* (**15**). From 2,5-furandicarboxylic acid. Conditions: 1.5 h reaction with NaHSe and 3 h reaction with methyl iodide. The solid was purified through column chromatography. A yellow solid was obtained. Yield: 30%; m.p.: 155–157 °C. ^1^H-NMR (CDCl_3_): δ 2.43 (s, 6H, –SeCH_3_), 7.21 ppm (s, 2H, H_3_ + H_4_). ^13^C-NMR (CDCl_3_): δ 5.0 (–SeCH_3_), 115.0 (C_3_ + C_4_), 153.7 (C_2_ + C_5_), 183.6 ppm (–C=O). IR (KBr): ν 2924 (w, C–H_aliph_), 1643 cm^−1^ (s, –C=O). MS [*m*/*z* (% abundance)]: 312 (179, M^+•^ +2), 217 (100), 189 (42), 133 (54), 94 (62), 66 (68). Elemental analysis calculated for C_8_H_8_O_3_Se_2_ (%): C: 30.97, H: 2.58; found: C: 31.09, H: 2.86.

#### 3.2.3. General Procedure for Compounds **1**–**3**, **5**, **7**, **10**, **12** and **13**

Under N_2_ atmosphere, absolute ethanol (10 mL) was added to a mixture of NaBH_4_ (2.15 mmol) and selenium (2 mmol) cooled by an ice bath, with magnetic stirring. Although reaction of these species occurs 1:1, a little excess of NaBH_4_ was added, due to the slow rate of decomposition of NaBH_4_ in this solvent reported by Klayman et al. [42] When the typical colorless solution of NaHSe was achieved, the ice bath was removed and the following reactions were carried out at room temperature. The acyl chloride was added and stirred for different amounts of time, depending on the reagents. Reaction was followed by TLC or IR. Before adding an excess of methyl iodide (1.5 mL), the mixture was filtered. After discoloration (20 min–1 h), the mixture was filtered, and ethanol was eliminated with rotatory evaporation or the product was precipitated with water. Compounds were purified through recrystallization from different solvents or column chromatography.

*Methyl 4-nitrobenzoselenoate* (**1**). From 4-nitrobenzoyl chloride. Conditions: 10 min reaction with NaHSe and 20 min reaction with methyl iodide. The compound was recrystallized from methylene chloride. A yellow powder was obtained. Yield: 28%; m.p.: 78–79 °C. ^1^H-NMR (CDCl_3_): δ 2.48 (s, 3H, –SeCH_3_), 8.07 (d, 2H, H_2_ + H_6_, *J*_2–3_ = *J*_6–5_ = 8.9 Hz), 8.33 ppm (d, 2H, H_3_ + H_5_, *J*_3–2_ = *J*_5–6_ = 8.9 Hz). ^13^C-NMR (CDCl_3_): δ 6.0 (–SeCH_3_), 124.1 (C_3_ + C_5_), 127.9 (C_2_ + C_6_), 143.3 (C_1_), 150.5 (C_4_), 193.7 ppm (–C=O). IR (KBr) ν 1665 (s, –C=O), 1518, 1348, 850 cm^−1^ (–NO_2_ arom). MS [*m/z* (% abundance)]: 245 (15, M^+•^ +1), 150 (100), 120 (24), 104 (94), 92 (45), 76 (61). Elemental analysis calculated for C_8_H_7_NO_3_Se (%): C: 39.36, H: 2.89, N: 5.74; found: C: 39.40, H: 3.17, N: 5.86.

*Methyl 4-methoxybenzoselenoate* (**2**). From 4-methoxybenzoyl chloride. Conditions: 1 h reaction with NaHSe and 20 min with methyl iodide. The compound was recrystallized from methylene chloride. A grayish powder was obtained. Yield: 23%; m.p.: 36–37 °C. ^1^H-NMR (CDCl_3_): δ 2.38 (s, 3H, –SeCH_3_), 3.88 (s, 3H, –OCH_3_), 6.94 (d, 2H, H_3_ + H_5_, *J*_3–2_ = *J*_5–6_ = 8.9 Hz), 7.91 ppm (d, 2H, H_2_ + H_6,_ *J*_2–3_ = *J*_6–5_ = 8.9 Hz). ^13^C-NMR (CDCl_3_): δ 5.0 (–SeCH_3_), 55.6 (–OCH_3_), 114.0 (C_3_ + C_5_), 129.4 (C_2_ + C_6_), 132.0 (C_1_), 164.0 (C_4_), 193.0 ppm (–C=O). IR (KBr): ν 2933–2839 (w, C–H_aliph_), 1652 (–C=O), 1264–1025 cm^−1^ (–OCH_3_). MS [*m/z* (% abundance)]: 230 (M^+•^ +1, 4), 135 (100), 107 (14), 92 (38), 77 (39), 63 (25). Elemental analysis calculated for C_9_H_10_O_2_Se (%): C: 47.18, H: 4.40; found: C: 47.38, H: 4.63.

*Methyl pyrazinecarboselenoate* (**3**). From pyrazinecarboxylic acid. Conditions: 30 min reaction with NaHSe and 30 min reaction with methyl iodide. The compound was recrystallized from hexane:methylene chloride (1:1). A yellow solid was obtained. Yield: 14%; m.p.: 48–49 °C. ^1^H-NMR (DMSO-*d*_6_): δ 2.28 (s, 3H, –SeCH_3_), 8.84 (dd, 1H, H_5_, *J*_5–6_ = 2.4, *J*_5–3_ = 1.5 Hz), 9.01 (d, 1H, H_6,_ *J*_6–5_ = 2.4), 9.03 ppm (d, 1H, H_3,_ *J*_3–5_ = 1.5 Hz). ^13^C-NMR (DMSO-*d*_6_): δ 4.9 (–CH_3_), 140.0 (C_2_), 145.8 (C_6_), 147.2 (C_5_), 150.9 (C_3_), 198.0 ppm (–C=O). IR (KBr): ν 2922 (w, C–H_aliph_), 1681 cm^−1^ (s, –C=O). MS [*m/z* (% abundance)]: 202 (M^+•^ +1, 37), 191 (45), 121 (61), 107 (100), 99 (95), 79 (80), 69 (61). Elemental analysis calculated for C_6_H_6_N_2_OSe (%): C: 35.84, H: 3.01, N: 13.93; found: C: 36.02, H: 3.31, N: 13.61. HRMS calculated for C_6_H_7_N_2_OSe^+^ [M + H]^+^ : 202.9718; found: 202.9778.

*Methyl 3-chlorothiophen-2-carboselenoate* (**5**). From 3-chlorothiophen-2-carboxylic acid. Conditions: 20 min reaction with NaHSe and 20 min reaction with methyl iodide. The compound was recrystallized from methylene chloride. A brownish powder was obtained. Yield: 31%; m.p.: 35–36 °C. ^1^H-NMR (DMSO-*d*_6_): δ 2.35 (s, 3H, –SeCH_3_), 7.33 (d, 1H, H_4_, *J*_4–5_ = 5.45 Hz), 8.10 ppm (d, 1H, H_5,_ *J*_5–4_ = 5.45 Hz). ^13^C-NMR (DMSO-*d*_6_): δ 6.9 (–SeCH_3_), 127.9 (C_4_), 131.6C_2_), 134.7 (C_5_), 137.2 (C_3_), 184.3 ppm (–C=O). IR (KBr): ν 3096 (w, C–H_arom_), 2938 (w, C–H_aliph_), 1620 cm^−1^ (s, –C=O). MS [*m/z* (% abundance)]: 240 (M^+•^ +1, 5), 211 (31), 145 (100), 43 (35). Elemental analysis calculated for C_6_H_5_ClOSSe (%): C: 30.08, H: 2.10; found: C: 30.35, H: 2.22.

*Methyl benzo[b]thiophene-2-carboselenoate* (**7**). From benzo[*b*]thiophene-2-carboxylic acid. Conditions: 30 min reaction with NaHSe and 20 min reaction with methyl iodide. The compound was precipitated with water and recrystallized from methylene chloride. Yield: 35%; m.p.: 65–67 °C. ^1^H-NMR (DMSO-*d*_6_): δ 2.41 (s, 3H, –SeCH_3_), 7.50 (ddd, 1H, H_6_, *J*_6–7_ = 8.0, *J*_6–4_ = 0.7 Hz), 7.57 (ddd, 1H, H_5_, *J*_5–4_ = 8.0, *J*_5–7_ = 1.2 Hz), 8.08 (d, 1H, H_4,_ *J*_4–6_ = 0.7 Hz, *J*_4–5_ = 8.0), 8.09 (d, 1H, H_7,_ *J*_7–5_ = 1.2 Hz), 8.41 ppm (s, 1H, H_3_). ^13^C-NMR (DMSO-*d*_6_): δ 6.3(–SeCH_3_), 124.1 (C_7_), 126.4 (C_5_), 127.4 (C_4_), 128.9 (C_6_), 130.6 (C_3_), 139.3 (C_3a_), 141.8 (C_7a_), 143.0 (C_2_), 187.6 ppm (–C=O). IR (KBr): ν 3064 (w, C–H_arom_), 2925 (w, C–H_aliph_), 1657 cm^−1^ (–C=O). MS [*m/z* (% abundance)]: 256 (M^+•^ +1, 6), 161 (100), 133 (42), 89 (65). Elemental analysis calculated for C_10_H_8_OSSe (%): C: 47.06, H: 3.16; found: C: 47.32, H: 3.19.

*Methyl 2-phenyl-4-quinolinecarboselenoate* (**10**). From 2-phenyl-4-quinolinecarboxylic acid. Conditions: 45 minute reaction with NaHSe cooled in the ice bath and 1 h reaction with methyl iodide at room temperature. The compound was precipitated with water and purified through column chromatography using methylene chloride as eluent. A white solid was obtained. Yield: 11%; m.p.: 119–120 °C. ^1^H-NMR (CDCl_3_): δ 2.57 (s, 3H, –SeCH_3_), 7.54–7.68 (m, 4H, H_2’_ + H_3’_ + H_5’_ + H_6’_), 7.84 (t, 1H, H_4’,_ *J*_4’–3’_ = *J*_4’–5’_ = 7.7 Hz), 8.23–8.25 (m, 2H, H_6_ + H_7_), 8.24 (s, 1H, H_3_), 8.33–8.41 ppm (m, 2H, H_5_ + H_8_). ^13^C-NMR (CDCl_3_): δ 7.3 (–SeCH_3_), 117.9 (C_3_), 121.4 (C_6_), 125.3 (C_5_), 128.2 (C_2’_ + C_6’_), 128.8 (C_3’_ + C_5’_), 129.5 (C_4’_), 129.8 (C_4a_), 130.7 (C_8_), 131.3 (C_7_), 138.2 (C_1’_), 146.3 (C_4_), 148.7 (C_8a_), 157.3 (C_2_), 197.1 ppm (–C=O). IR (KBr): ν 3056 (w, C–H_arom_), 2922 (w, C–H_aliph_), 1685 cm^−1^ (s, –C=O). MS [*m/z* (% abundance)]: 327 (M^+•^ +1, 5), 232 (96), 204 (100), 75 (33). Elemental analysis calculated for C_17_H_13_NOSe (%): C: 62.58, H: 4.02, N: 4.29; found: C: 62.58, H: 3.89, N: 4.28. HRMS calculated for C_17_H_14_NOSe^+^ [M + H]^+^: 328.0235; found: 328.0251.

*Methyl 2-(4-pyridyl)thiazole-4-carboselenoate* (**12**). From 2-(4-pyridyl)thiazole-4-carboxylic acid. Conditions: 30 minute reaction with NaHSe cooled on an ice bath and 30 minute reaction with methyl iodide at room temperature. The compound was recrystallized from methylene chloride and diethyl ether (1:1). A white solid was obtained. Yield: 20%. ^1^H-NMR (CDCl_3_): δ 2.40 (s, 3H, –SeCH_3_), 8.00 (d, 2H, H_2’_ + H_6’_, *J*_2’–3’_ = *J*_6’–5’_ = 6.2 Hz), 8.20 (s, 1H, H_5_), 8.82 ppm (d, 2H, H_3’_ + H_5’,_ *J*_3’–2’_ = *J*_5’–6’_ = 6.2 Hz). ^13^C-NMR (CDCl_3_): δ 5.3 (–SeCH_3_), 121.4 (C_2’_ + C_6’_), 122.7 (C_5_), 141.1 (C_1’_), 149.6 (C_3’_ + C_5’_), 156.5 (C_4_), 164.9 (C_2_), 189.7 ppm (–C=O). IR (KBr): ν 3134 (w, C–H_arom_), 2919 (w, C–H_aliph_), 1665 (s, C=O), 1132 cm^−1^ (s, thiazole ring vibration). MS [*m/z* (% abundance)]: 284 (M^+•^ +1, 21), 189 (84), 156 (100), 128 (77), 57 (91), 69 (59). Elemental analysis calculated for C_10_H_8_N_2_OSSe: C: 42.41, H: 2.85, N: 9.89; found: C: 42.14, H: 3.34, N: 9.84. HRMS calculated for C_10_H_9_N_2_OSSe^+^ [M + H]^+^: 284.9595; found: 284.9578.

*Methyl 9-acridinecarboselenoate* (**13**). From 9-acridinecarboxylic acid. Before adding the chloride to the reaction, it was dissolved in dry chloroform, treated with triethylamine (1:1) for 20 min to eliminate the hydrochloride and then used for reaction without further treatment. Conditions: 25 min reaction with NaHSe and 25 min reaction with methyl iodide. The compound was recrystallized from hexane. An orange solid was obtained. Yield: 23%; m.p.: 132–134 °C. ^1^H-NMR (CDCl_3_): δ 2.64 (s, 3H, –SeCH_3_), 7.58–7.63 ppm (dd, 2H, H_2_ + H_7,_ *J*_2–3_ = *J*_7–6_ = 7.7, *J*_2–1_ = *J*_7–8_ = 8.7), 7.79–7.85 (dd, 2H, H_3_ + H_6_, *J*_3–2_ = *J*_6–7_ = 7.7; *J*_3–4_ = *J*_6–5_ = 8.8), 8.09 (d, 2H, H_1_ + H_8,_ *J*_1–2_ = *J*_8–7_ = 8.7), 8.31 ppm (d, 2H, H_4_ + H_5,_ *J*_4–3_ = *J*_5–6_ = 8.8). ^13^C-NMR (CDCl_3_): δ 6.9 (–SeCH_3_), 120.2 (C_8a_ + C_9a_), 120.2 (C_1_ + C_8_), 124.6 (C_2_ + C_7_), 126.9 (C_4_ + C_5_), 129.2 (C_3_ + C_6_), 130.7 (C_9_), 148.1 (C_4a_ + C_10a_), 198.4 ppm (–C=O). IR (KBr): ν 2971–2925 (w, C–H_aliph_), 1673 cm^−1^ (s, –C=O). MS [*m/z* (% abundance)]: 301 (M^+•^ +1, 4), 206 (100), 178 (93), 151 (31). Elemental analysis calculated for C_15_H_11_NOSe (%): C: 60.01, H: 3.69; N: 4.67; found: C: 59.83, H: 3.97, N: 4.38. HRMS calculated for C_15_H_12_NOSe^+^ [M + H]^+^: 302.0078; found: 302.0091.

### 3.3. Methylselenol Release

A stock solution of the compounds was prepared in anhydrous DMSO (10 mM). The compounds were placed in a 96-well plate in a final concentration of 100 μM. Reaction started after adding 200 μL of an aqueous 100 mM Na_2_HPO_4_ buffer (1 mM EDTA, pH = 8), containing 100 μM of DTNB from a freshly prepared 10 mM ethanolic stock, which were added just before starting the reaction. The DTNB concentration was quantified with a cysteine standard curve (7.5–100 μM). Blanks for the compounds in the absence of DTNB were also included as well as a blank to measure the slow but spontaneous hydrolysis of DTNB, which was further subtracted from all the values. Absorbance was read at 412 nm in a FLUOstar Omega microplate reader (BMG LabTech, Ortenberg, Germany). Results represent means of triplicate values ± SD. 

### 3.4. Theoretical Calculations of Molecular Properties

The drug-likeness score along with the TPSA values and the properties described in Lipinski’s Rule of Five [molecular weight (MW) ≤ 500 Da, logP ≤ 5, H-bond donors (nOHNH) ≤ 5 and H-bond acceptors (nON) ≤ 10] were calculated using the freely download version of Osiris DataWarrior v.4.5.2 [53] and the online available Molinspiration [54] property calculation programs, respectively. Likewise, the toxicity risks (mutagenic, tumorigenic, irritant and reproductive effects) were obtained by Osiris DataWarrior program and are labeled in different colours (green for no risk, orange for mild risk and red for high risk).

### 3.5. Biology

#### 3.5.1. Radical Scavenging Assays

##### DPPH assay

The assay was performed following a described protocol [41], with few modifications. Briefly, a stock solution of DPPH**^·^** (2,2-diphenyl-1-picrylhydrazyl, Sigma Aldrich, Madrid, Spain) in methanol (0.04 mg/mL) was freshly prepared. Absorbance of the stock solution was adjusted at 0.8 ± 0.02 at 516 nm for each experiment. Due to solubility issues in methanol, a stock solution of each compound (10 mM) was prepared in DMSO. Compounds were tested at a final concentration of 100 μM. The negative control contained the same amount of DMSO to avoid interferences. The reaction was incubated in a 2 mL microtube in the dark and 300 μL were seeded in a 96-well plate at the determined times, to avoid erratic measures due to methanol evaporation if incubated all the time in the plate. Discolouration to the yellowish reduced form was followed at 516 nm in a FLUOstar Omega (BMG LabTech) plate reader. Ascorbic acid was used as a positive control. 

Percentage of scavenged DPPH^·^ was calculated as follows:

$$\%\;of\;scavenged\;DPPH^{\cdot }=100\;\times \;\frac{A_{\mathrm{control}}-A_{\mathrm{sample}}}{A_{\mathrm{control}}}$$

with A_control_ being the absorbance of the negative control (only DMSO) and A_sample_ the absorbance of each tested compound. The experiment was performed three times in quadruplicate. 

##### ABTS assay

ABTS assay was performed with a colorimetric assay [55]. Briefly, ABTS was dissolved (1 mg/mL) in a 2.45 mM potassium persulfate solution and kept overnight in the dark at room temperature to generate the radical ABTS^+**·**^. For the reaction, the mixture was diluted with 50% ethanol to an absorbance of 0.700 ± 0.02 at 741 nm. The compounds were dissolved in DMSO and tested at different concentrations. The negative control had the same amount of DMSO. Ascorbic acid was used as a positive control. The reaction was started after adding 180 μL of the solution. Absorbance was read with a FLUOstar Omega (BMG LabTech) plate reader after 6 min incubation in the dark. The percentage of scavenged ABTS^+**·**^ was calculated with the following formula:

$$\%\;of\;scavenged\;ABTS^{+\cdot }=100\;\times \;\frac{A_{\mathrm{control}}-A_{\mathrm{sample}}}{A_{\mathrm{control}}}$$

where A_control_ corresponds to the absorbance of the negative control (DMSO only) and A_sample_ is the absorbance of the tested compounds. The experiment was performed in quadruplicate.

#### 3.5.2. Cell Culture

All cells were purchased from the American Type Culture Collection (ATCC, Barcelona, Spain). HT-29 (colorectal adenocarcinoma), HTB-54 (grade III lung carcinoma), MCF7 (mammary adenocarcinoma), PC-3 (grade IV prostate adenocarcinoma) and K-562 (chronic myelogenous leukemia) were cultured in RPMI (Gibco, Madrid, Spain), 10% FBS (Gibco), 100 units/mL penicillin and 100 μg/mL streptomycin (Gibco). BEAS-2B cell line (normal epithelial lung) was cultured in DMEM (Gibco), 10% FBS, 100 units/mL penicillin and 100 μg/mL streptomycin. 184B5 (normal mammary gland) cell line was cultured in DMEM:F12 supplemented with 5% FBS, 1 × ITS (Lonza, Barcelona, Spain), 100 nM hydrocortisone (Sigma-Aldrich), 2 mM sodium pyruvate (Lonza), 20 ng/mL EGF (Sigma-Aldrich), 0.3 nM *trans*-retinoic acid (Sigma-Aldrich), 100 units/mL penicillin and 100 µg/mL streptomycin. Cells were cultured at 37 °C under 5% CO_2_.

#### 3.5.3. Viability Assay

Cell viability after treatment was assessed by the well-known 3-(4,5-dimethyl-thiazol-2-yl)-2,5-diphenyltetrazolium bromide (MTT, Sigma-Aldrich) assay [21]. Briefly, 10,000 cells were seeded in 96-well plates, treated and incubated for 72 h. Dilutions of the compounds in cell medium were freshly prepared from a 0.01 M stock in DMSO. After treatment, 50 μL of MTT solution in PBS (2 mg/mL) were added and cells were incubated for 4 h. Medium was removed and 150 μL of DMSO was added to dissolve the formed formazan crystals. Absorbance was read at 550 nm in a microplate absorbance reader (Sunrise, Tecan, Männedorf, Switzerland).

#### 3.5.4. Cell Cycle and Cell Death Analysis

3 × 10^6^ or 2 × 10^6^ MCF7 cells were seeded in 25 cm^2^ flasks for treatments up to 24 h or 48 h, respectively. DMSO (vehicle) or the dissolved compounds were added 24 h after seeding and cells were incubated for different times at 37 °C under 5% CO_2_. Cell cycle and cell death were analyzed simultaneously with the Apo-Direct kit (BD Pharmingen, Madrid, Spain), following the manufacturer’s protocol. Briefly, cells were fixed in 1% paraformaldehyde for 45 min at 0 °C, washed with PBS and incubated for at least 30 min in 70% ethanol on ice. Cells were then stained both with FITC-dUTP (1 h, 37 °C) and propidium iodide (30 min, room temperature) and analyzed by flow cytometry using a Coulter Epics XL cytometer (Beckman Coulter, Brea, CA, USA). 

#### 3.5.5. Caspase and Autophagy Inhibitors Assay

2 × 10^6^ MCF7 cells were seeded in 25 cm^2^ flasks. After 24 h, cells were pre-treated with 50 μM of the pan-caspase inhibitor Z-VAD-FMK (BD Pharmingen) or 100 nM wortmannin (Santa Cruz Biotechnology, Heidelberg, Germany) for 1 h before co-incubating them with 25 μM of compound **5**, 30 μM of compound **15** or vehicle (maximum amount of DMSO used in the treatments) for 48 h. Cells were collected and processed with the Apo-Direct kit as described previously.

#### 3.5.6. Enzymatic Assays

##### Thioredoxin Reductase Activity Assay

The assay was performed as previously described [56], but in a final volume of 200 μL in a 96-well plate. Reaction started after addition of 133 μL of TE buffer (20 mM Tris, 2 mM EDTA, pH = 8), 0.227 mM NADPH (freshly prepared) to different concentrations of compounds solved in DMSO and TrxR1 (final concentration 100 nM). NADPH consumption was followed measuring absorbance decrease at 340 nm in a VersaMax microplate reader (Molecular Devices, Sunnyvale, CA, USA) over 45 min. A background sample with DMSO without the compounds was included and subtracted from the values.

##### Glutaredoxin/Glutathione Assay

The assay was performed as previously described [57], with minor modifications and adapted for a 96-well plate. A mixture of 0.1 M Tris pH 8, 2 mM EDTA pH = 8, 0.1 mg/mL BSA, 1 mM GSH, 200 μM NADPH, and 0.008 OD/mL yeast GR was prepared (all reagents were purchased from Sigma-Aldrich). Reactions were performed with 1 μM hGrx1 (IMCO Corporation, Stockholm, Sweden) when corresponding and different concentrations of the compounds. A 100 μL mixture was added, and the final volume was adjusted to 110 μL per well. Consumption of NADPH was monitored at A_340_ on a VersaMax microplate reader (Molecular Devices) for 45 min.

#### 3.5.7. Statistics

The statistical analyses were performed with a Mann-Whitney test or a Wilcoxon test for the inhibitors assay. Statistical significance was calculated using GraphPad Prism 6.01. (* *p* < 0.05, ** *p* < 0.01, *** *p* < 0.001).

## 4. Conclusions

A novel series of 15 methylselenoesters has been synthesized. Differences in the hydrolytic release of CH_3_SeH in aqueous medium indicated that the chemical features of the core of the molecule modulated the lability of the carbonyl-Se bond. In the preliminary assessment of redox properties, only compound **11** presented radical scavenging activity. The compounds were able to inhibit proliferation in different cancer cell lines and their toxicity towards non-malignant cell lines was in the same range as MSA. Compounds **5** and **15** arrested cell cycle in G_2_/M phase in MCF7 cell line. Although both compounds induced cell death in a dose-dependent manner, only compound 15 was time-dependent. Pre-treatment of the cells with a pan-caspase inhibitor partially prevented cell death, suggesting that the caspase pathway is implicated in their mechanism. Even though further research is needed, results suggest that these compounds might be good precursors of CH_3_SeH, one of the key metabolites in Se anticancerous activity and might be of great interest as potential antitumor agents.

## Figures and Tables

**Table 1:** Structure of the novel synthesized methylselenoesters.

**General Structure** 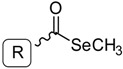					
**Ref. ^a^**	**R**	**Solvent ^b^**	**Ref.**	**R**	**Solvent**
**1**	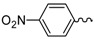	EtOH	**8**	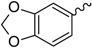	H_2_O
**2**	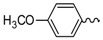	EtOH	**9**	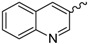	H_2_O
**3**	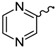	EtOH	**10**	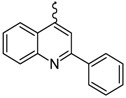	EtOH
**4**	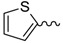	H_2_O			
**5**	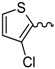	EtOH	**11**	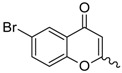	H_2_O
**6**	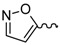	H_2_O	**12**	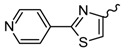	EtOH
**7**	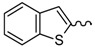	EtOH	**13**	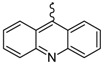	EtOH
**General structure**		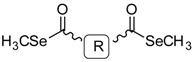			
**Ref.**	**R**	**Solvent**	**Ref.**	**R**	**Solvent**
**14**	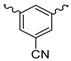	H_2_O	**15**	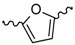	H_2_O

The wavy bond indicates the attachment position for the methylselenoester moiety. ^a^ Compound reference; ^b^ Solvent used as reaction medium.

**Table 2:** Theoretical calculations of molecular properties based in Molinspiration calculations.

	Molinspiration Calculations							
Compound	milogP ^a^	PSA ^b^	MW ^c^	nON ^d^	nOHNH ^e^	nViolations ^f^	Nrotb ^g^	Volume
**1**	2.39	62.90	244.11	4	0	0	3	166.41
**2**	2.48	26.30	229.14	2	0	0	3	168.62
**3**	0.96	42.85	201.09	3	0	0	2	134.77
**4**	2.33	17.07	205.14	1	0	0	2	133.79
**5**	2.93	17.07	239.59	1	0	0	2	147.33
**6**	1.15	43.10	190.09	3	0	0	2	120.49
**7**	3.63	17.07	255.20	1	0	0	2	177.78
**8**	2.32	35.54	243.12	3	0	0	2	167.01
**9**	2.41	29.96	250.16	2	0	0	2	182.91
**10**	4.55	29.96	326.26	2	0	0	3	254.32
**11**	3.33	47.28	346.04	3	0	0	2	205.51
**12**	2.23	42.85	283.21	3	0	0	3	196.89
**13**	4.19	29.96	300.22	2	0	0	2	226.91
**14**	2.60	57.93	345.12	3	0	0	4	218.97
**15**	2.41	47.28	310.07	3	0	0	4	183.68
**MSA**	0.60	37.30	127.00	2	1	0	0	68.78

^a^ Octanol/water partition coefficient; ^b^ Molecular Polar Surface Area; ^c^ Molecular weight; ^d^ H-bond acceptors; ^e^ H-bond donors; ^f^ Number of violations of Lipinski’s Rule of Five; ^g^ Number of rotatable bonds.

**Table 3:** Predicted toxicity of the compounds according to Osiris DataWarrior-based calculations.

Compound	Toxicity Level				Osiris Calculations
	Mutagenic	Tumorigenic	Irritant	RE ^a^	DL ^b^
**1**					−10.80
**2**					−5.34
**3**					−5.25
**4**					−1.96
**5**					−2.77
**6**					−5.52
**7**					−2.67
**8**					−2.91
**9**					−5.25
**10**					−5.25
**11**					−4.86
**12**					−0.85
**13**					−5.25
**14**					−9.53
**15**					−2.76
**MSA**					−5.69

^a^ Reproductive effects; ^b^ Drug-likeness. 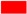 High risk; 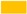 Mild risk; 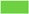 No risk.

**Table 4:** Cytotoxic activity of the novel methylselenoesters at 72 h. Average GI_50_, TGI and LC_50_ ± SD values are expressed in μM.

	**Cell Line**											
**No.**	**PC-3 ^a^**			**MCF7 ^b^**			**184B5 ^c^**					
	**GI_50_**	**TGI**	**LC_50_**	**GI_50_**	**TGI**	**LC_50_**	**GI_50_**	**TGI**	**LC_50_**			
**1**	5.8 ± 0.7	10.4 ± 2.3	75.7 ± 3.3	4.1 ± 0.5	7.9 ± 0.9	45.0 ± 4.6	3.4 ± 0.4	6.4 ± 1.1	9.3 ± 0.6			
**2**	7.3 ± 1.7	45.1 ± 4.5	>100	4.1 ± 0.8	9.3 ± 4.1	74.8 ± 4.0	3.7 ± 0.8	6.4 ± 1.5	9.0 ± 2.1			
**3**	5.4 ± 1.1	15.3 ± 1.7	60.5 ± 4.3	3.5 ± 0.8	7.2 ± 1.0	40.1 ± 4.4	3.1 ± 0.5	6.6 ± 0.9	17.6 ± 4.4			
**4**	5.2 ± 0.9	19.4 ± 3.6	62.0 ± 4.9	5.3 ± 1.4	22.8 ± 3.8	89.6 ± 4.1	3.6 ± 0.8	7.2 ± 1.4	27.9 ± 4.6			
**5**	8.6 ± 1.7	47.7 ± 2.9	91.6 ± 3.9	3.1 ± 0.9	6.9 ± 1.1	35.9 ± 4.4	3.5 ± 0.4	5.9 ± 0.3	8.3 ± 0.2			
**6**	4.5 ± 1.3	7.7 ± 0.1	61.5 ± 14.0	3.5 ± 1.0	8.8 ± 0.7	71.9 ± 3.2	2.7 ± 0.2	6.0 ± 0.4	9.3 ± 2.0			
**7**	18.4 ± 4.5	47.8 ± 3.9	77.3 ± 4.5	6.9 ± 1.4	61.6 ± 4.1	>100	3.9 ± 1.2	6.9 ± 1.7	9.9 ± 0.8			
**8**	5.7 ± 1.6	11.0 ± 3.1	96.0 ± 4.7	4.6 ± 0.5	9.2 ± 1.3	68.7 ± 2.5	3.7 ± 0.3	6.4 ± 0.2	9.0 ± 0.4			
**9**	5.9 ± 0.5	10.0 ± 1.9	84.1 ± 8.9	4.4 ± 0.8	8.1 ± 1.2	48.0 ± 7.5	3.7 ± 0.5	6.2 ± 0.4	8.6 ± 0.4			
**10**	4.4 ± 1.3	9.9 ± 4.7	70.9 ± 5.2	5.6 ± 1.3	51.3 ± 3.7	>100	3.9 ± 0.7	6.3 ± 0.5	8.7 ± 0.4			
**11**	5.3 ± 1.1	17.1 ± 5.0	66.2 ± 2.4	4.2 ± 0.7	8.2 ± 1.1	>100	3.3 ± 0.1	6.7 ± 0.9	14.5 ± 4.5			
**12**	16.9 ± 4.1	50.9 ± 4.9	85.0 ± 4.7	4.4 ± 1.0	8.1 ± 1.7	56.2 ± 9.6	3.4 ± 0.3	6.5 ± 0.8	9.6 ± 2.8			
**13**	39.7 ± 5.1	>100	>100	4.2 ± 1.8	35.4 ± 4.7	83.4 ± 4.1	6.6 ± 1.2	26.8 ± 1.0	65.0 ± 4.5			
**14**	7.2 ± 3.5	32.3 ± 3.7	68.7 ± 3.0	4.9 ± 0.8	8.4 ± 1.1	54.4 ± 2.7	3.8 ± 0.7	6.5 ± 0.6	9.3 ± 0.7			
**15**	4.8 ± 0.8	8.4 ± 0.6	47.8 ± 4.4	1.8 ± 0.9	7.2 ± 1.9	57.0 ± 4.0	3.5 ± 1.1	6.4 ± 0.5	9.2 ± 0.9			
**MSA**	4.4 ± 1.1	8.5 ± 2.4	47.6 ± 4.6	1.5 ± 0.5	5.4 ± 0.5	9.4 ± 1.0	5.3 ± 1.2	9.2 ± 1.3	13.1 ± 1.3			
	**Cell Line**											
**No.**	**K-562 ^d^**			**HT-29 ^e^**			**HTB-54 ^f^**			**BEAS-2B ^g^**		
	**GI_50_**	**TGI**	**LC_50_**	**GI_50_**	**TGI**	**LC_50_**	**GI_50_**	**TGI**	**LC_50_**	**GI_50_**	**TGI**	**LC_50_**
**1**	50.1 ± 2.3	85.3 ± 4.9	>100	5.0 ± 0.7	9.3 ± 1.4	87.2 ± 5.0	22.5 ± 4.5	50.5 ± 5.1	78.6 ± 2.9	3.8 ± 0.3	6.4 ± 0.4	9.0 ± 0.7
**2**	40.8 ± 4.8	70.6 ± 5.9	>100	4.1 ± 0.1	7.5 ± 1.1	50.0 ± 2.4	6.8 ± 1.7	29.7 ± 4.3	70.0 ± 2.9	3.2 ± 0.5	5.8 ± 0.6	8.4 ± 1.0
**3**	30.2 ± 2.2	59.1 ± 2.1	87.9 ± 2.0	4.7 ± 0.6	9.1 ± 0.8	>100	6.2 ± 1.5	21.1 ±4.1	71.1 ± 3.8	3.6 ± 0.6	6.1 ± 0.5	8.6 ± 0.5
**4**	40.6 ± 4.5	74.6 ± 4.9	>100	3.4 ± 0.2	7.2 ± 1.2	47.5 ± 3.2	8.5 ± 3.1	39.0 ± 3.1	73.9 ± 4.1	2.9 ± 0.3	5.8 ± 0.7	8.7 ± 2.1
**5**	38.7 ± 2.1	67.0 ± 1.9	95.2 ± 2.2	3.6 ± 0.3	6.3 ± 0.2	8.9 ± 0.2	4.9 ± 0.2	9.3 ± 2.7	61.5 ± 3.2	2.7 ± 0.5	5.3 ± 0.7	8.0 ± 1.0
**6**	18.5 ± 7.1	58.3 ± 3.5	98.1 ± 0.7	5.0 ± 0.8	15.1 ± 6.7	90.8 ± 15.0	9.0 ± 2.8	43.9 ± 3.9	82.0 ± 1.3	3.0 ± 0.9	5.6 ± 0.7	8.2 ± 0.5
**7**	29.2 ± 4.4	58.8 ± 1.5	88.3 ± 3.3	3.7 ± 0.2	7.4 ± 0.9	41.8 ± 4.5	17.5 ± 4.2	55.4 ± 4.4	93.4 ± 4.8	3.9 ± 0.6	6.8 ± 0.8	9.8 ± 1.1
**8**	29.7 ± 4.7	62.6 ± 3.9	95.4 ± 4.9	5.9 ± 0.8	27.7 ± 4.5	81.1 ± 4.0	6.0 ± 0.9	23.2 ± 2.6	77.1 ± 4.9	3.1 ± 0.3	5.6 ± 0.6	8.0 ± 1.1
**9**	39.5 ± 3.1	72.6 ± 4.1	>100	3.4 ± 0.2	7.0 ± 0.2	37.2 ± 5.0	7.8 ± 1.2	33.0 ± 4.5	69.2 ± 2.7	4.1 ± 1.0	7.1 ± 4.5	15.3 ± 9.1
**10**	9.7 ± 4.9	59.1 ± 3.5	>100	4.1 ± 0.3	7.5 ± 0.7	35.0 ± 2.4	5.9 ± 1.1	16.7 ± 2.3	64.4 ± 4.2	3.1 ± 0.5	5.5 ± 0.4	7.8 ± 0.5
**11**	81.9 ± 4.0	>100	>100	4.9 ± 0.7	9.6 ± 3	74.4 ± 4.2	9.4 ± 3.5	52.3 ± 2.9	>100	2.4 ± 0.9	5.0 ± 0.8	7.6 ± 0.7
**12**	82.0 ± 4.4	>100	>100	5.1 ± 0.1	14.2 ± 6.6	>100	9.8 ± 5.1	44.9 ± 3.7	80.5 ± 3.5	3.7 ± 1.3	6.4 ± 1.3	9.2 ± 1.0
**13**	n.a. ^h^	n.a.	n.a.	6.0 ± 0.5	16.5 ± 1.9	66.6 ± 0.1	n.a.	n.a.	n.a.	3.8 ± 0.1	6.4 ± 0.2	8.9 ± 0.4
**14**	29.9 ± 0.6	54.7 ± 0.7	79.5 ± 0.7	3.8 ± 0.1	7.9 ± 0.7	>100	3.8 ± 0.6	7.7 ± 1.0	66.5 ± 4.5	3.2 ± 0.7	5.5 ± 0.5	7.8 ± 0.3
**15**	42.0 ± 3.8	70.6 ± 3.4	99.2 ± 5.3	3.0 ± 0.5	6.6 ± 0.4	35.8 ± 5.0	15.6 ± 4.3	46.0 ± 3.0	76.3 ± 1.1	2.6 ± 0.8	5.6 ± 0.9	8.6 ± 1.2
**MSA**	n.a.	n.a.	n.a.	n.a.	n.a.	n.a.	3.5 ± 0.1	6.9 ± 0.2	19.9 ± 1.2	3.7 ± 0.7	6.2 ± 1.2	8.7 ± 1.8

^a^ Prostate adenocarcinoma; ^b^ Mammary adenocarcinoma; ^c^ Non-malignant mammary epithelium; ^d^ Chronic myelogenous leukemia; ^e^ Colorectal adenocarcinoma; ^f^ Lung carcinoma; ^g^ Non-malignant bronchial epithelial; ^h^ Data not available.

## Supplementary Materials

Supplementary materials are available online. ^1^H- and ^13^C-NMR of all the new synthesized compounds.
