# Supplementary material for: Novel Methylselenoesters as Antiproliferative Agents
Source: Molecules. 2017 Aug 2;22(8):1288. doi: 10.3390/molecules22081288 (PMC6152192; doi:10.3390/molecules22081288)
Supplement: Supplementary file 1 [file molecules-22-01288-s001.pdf]

Supplementary material

Novel methylselenoesters as antiproliferative agents

Nuria Díaz-Argelich[1, 2, 3], Ignacio Encío[4], Daniel Plano[1, 2], Aristi P. Fernandes[3], Juan Antonio Palop[1, 2], Carmen Sanmartín[1, 2*]

1) University of Navarra. Faculty of Pharmacy and Nutrition. Department of Organic and Pharmaceutical Chemistry, Irunlarrea 1, 3E-1008 Pamplona, Spain
2) Oncology and Hematology Section, IdiSNA, Navarra Institute for Health Research, Irunlarrea 3, E-31008, Pamplona, Spain
3) Division of Biochemistry, Department of Medical Biochemistry and Biophysics (MBB), Karolinska Institutet, SE-171 77 Stockholm, Sweden
4) Department of Health Sciences, Public University of Navarra, Avda. Barañain s/n, E-31008 Pamplona, Spain

Contents:

Representative spectra (^{1}H and ^{13}C NMR) of the methylselenoesters

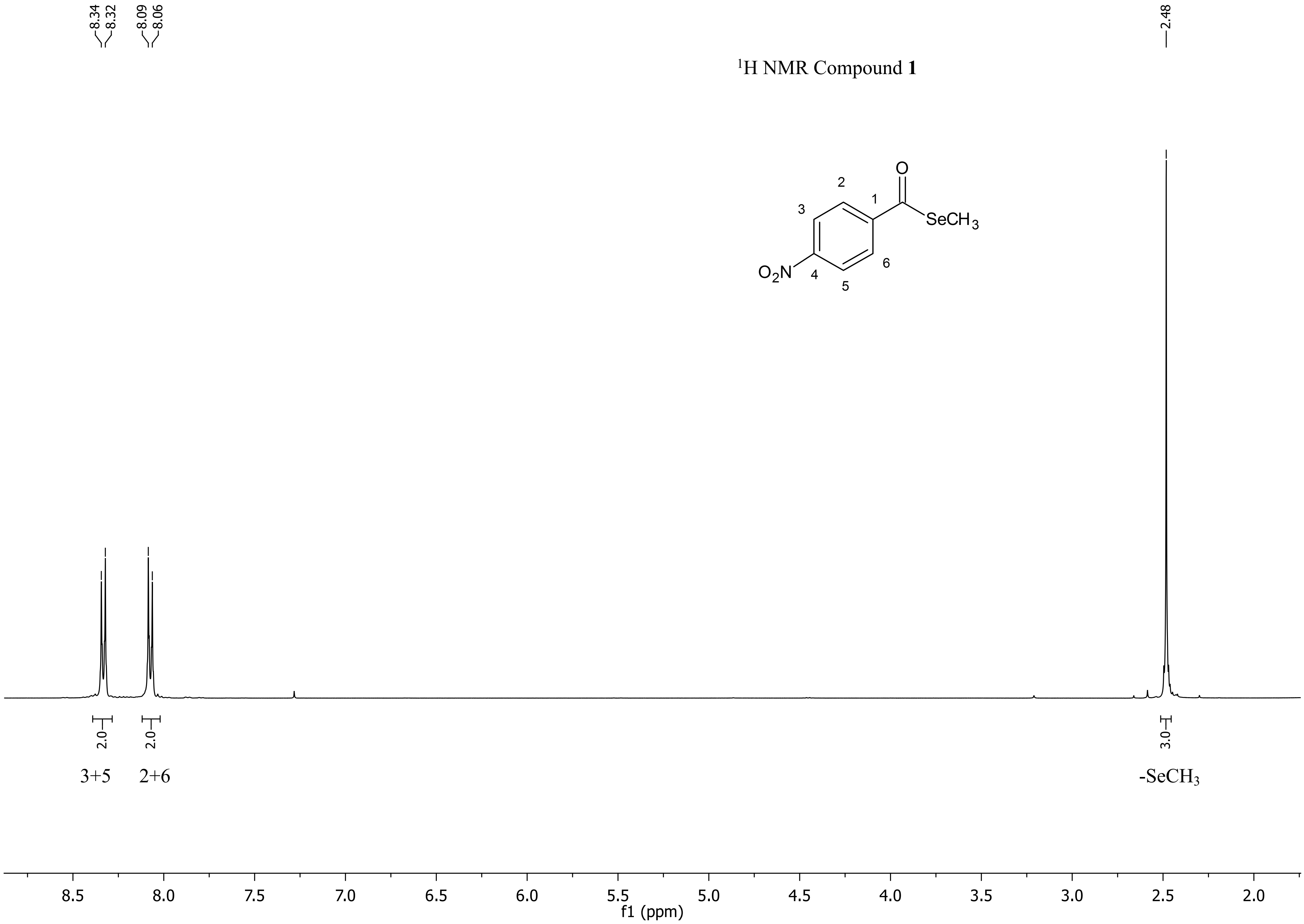
8.34
8.32
8.09
8.06
2.48
1H NMR Compound 1
O
2
1
3
SeCH3
6
O2N
4
5
2.0
2.0
3.0
3+5
2+6
-SeCH3
8.5
8.0
7.5
7.0
6.5
6.0
5.5
5.0
4.5
4.0
3.5
3.0
2.5
2.0
f1 (ppm)

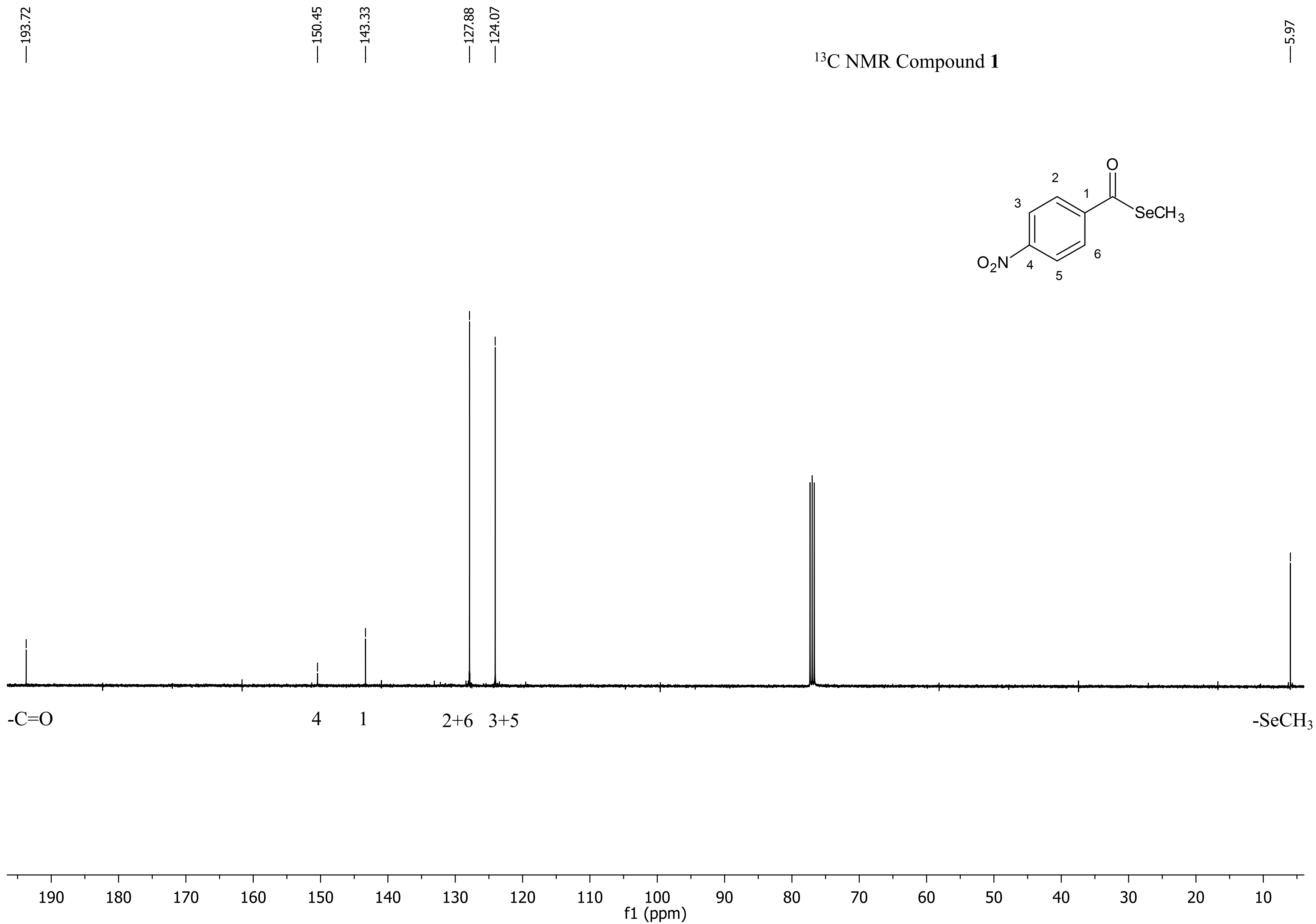

193.72
150.45
143.33
127.88
124.07
5.97
13C NMR Compound 1
O
2
1
3
SeCH3
O2N
4
6
5
-C=O
4
1
2+6
3+5
-SeCH3
190
180
170
160
150
140
130
120
110
100
90
80
70
60
50
40
30
20
10
f1 (ppm)

^{1}H NMR Compound **2**

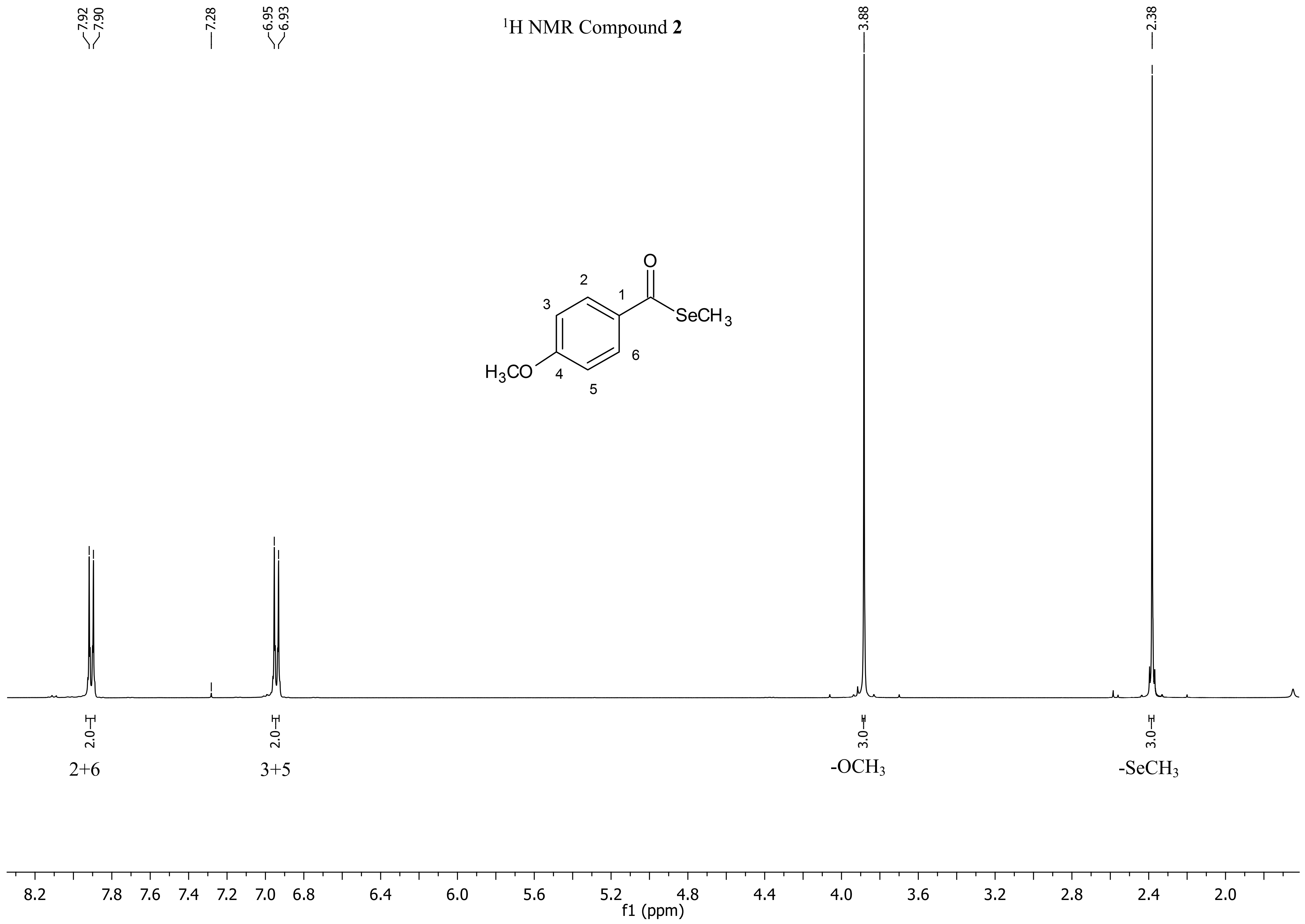

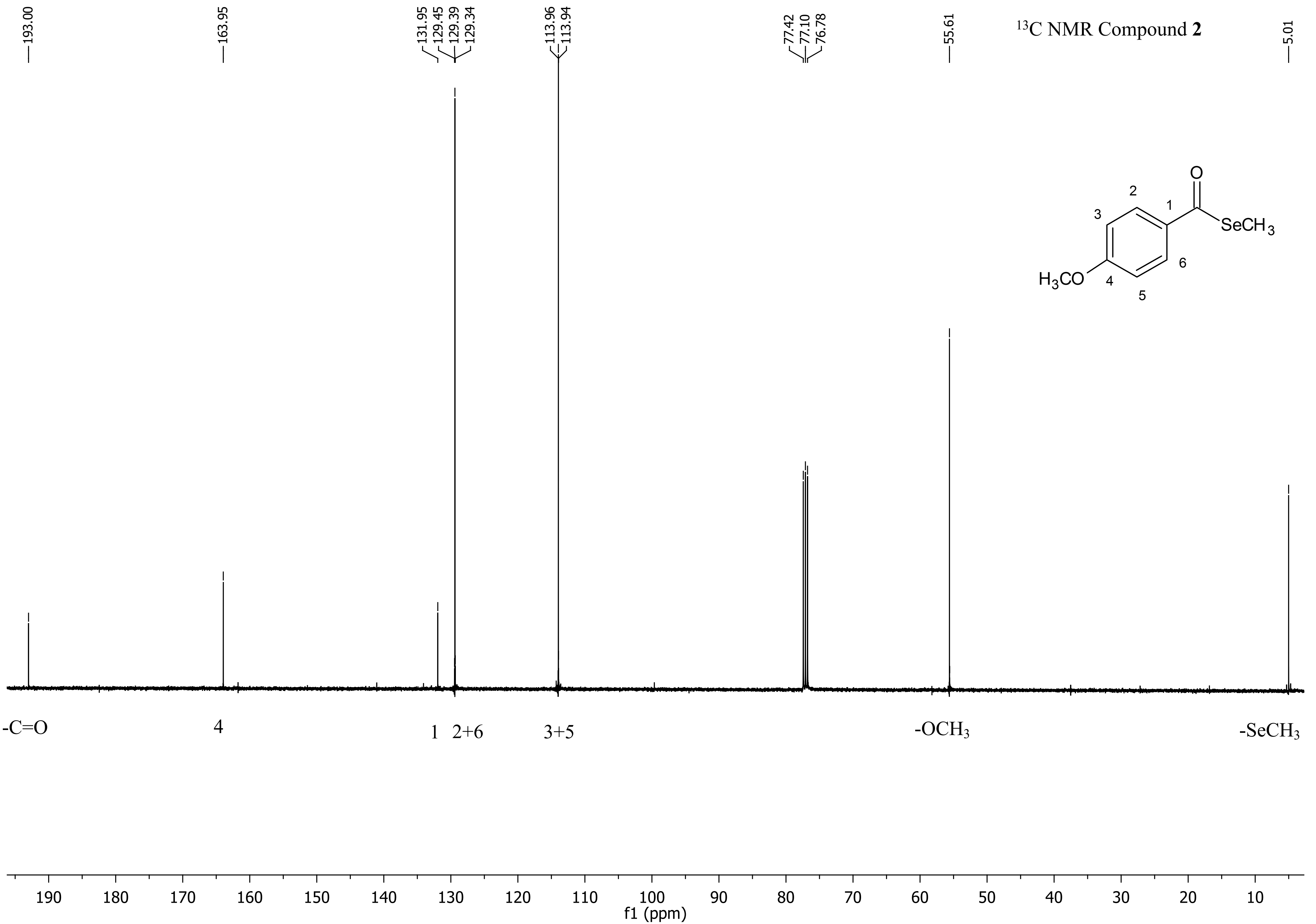

193.00
163.95
131.95
129.45
129.39
129.34
113.96
113.94
77.42
77.10
76.78
55.61
5.01
13C NMR Compound 2
O
SeCH3
H3CO
1
2
3
4
5
6
-C=O
4
1 2+6
3+5
-OCH3
-SeCH3
190 180 170 160 150 140 130 120 110 100 90 80 70 60 50 40 30 20 10
f1 (ppm)

^{1}H NMR Compound **3**

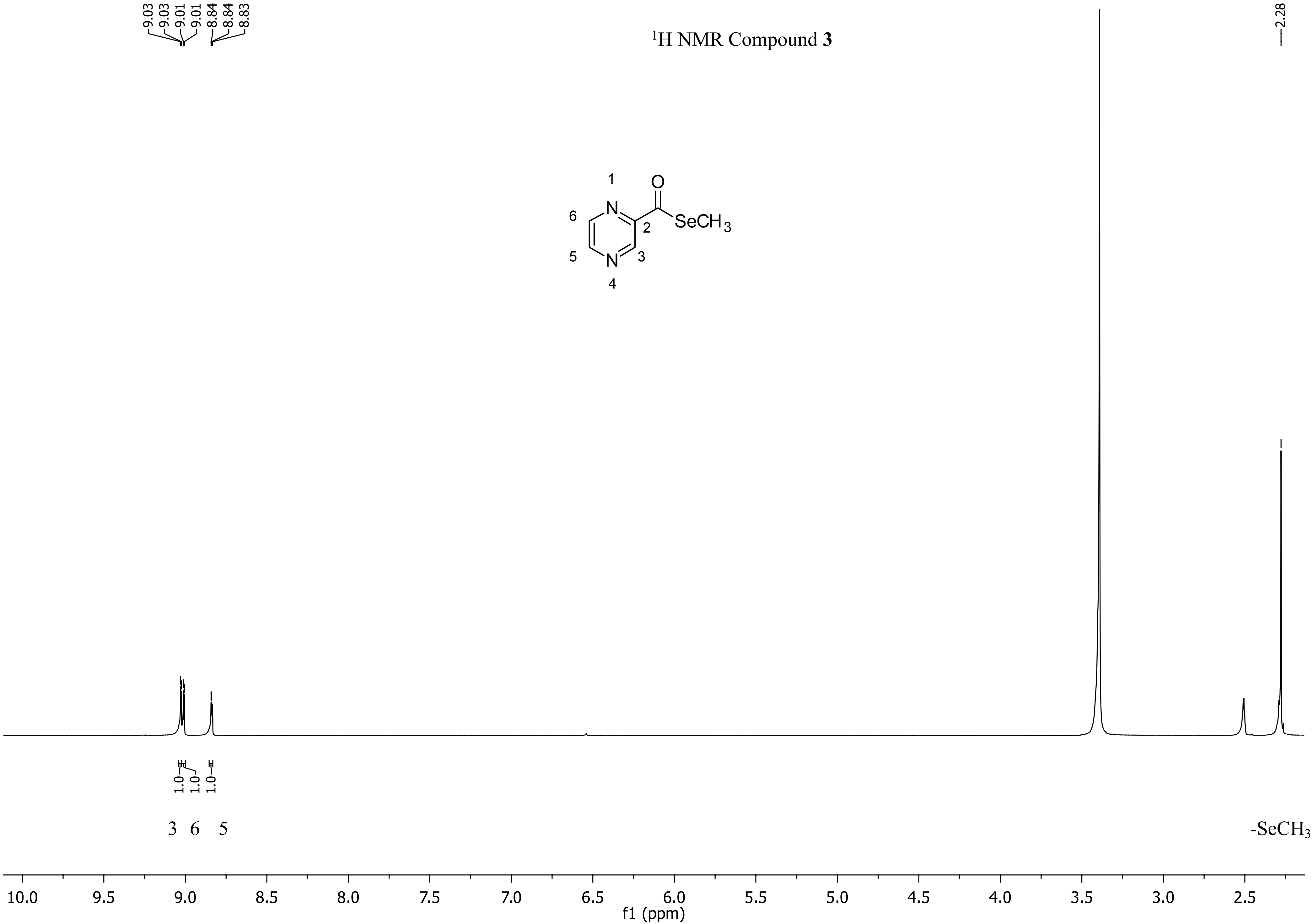

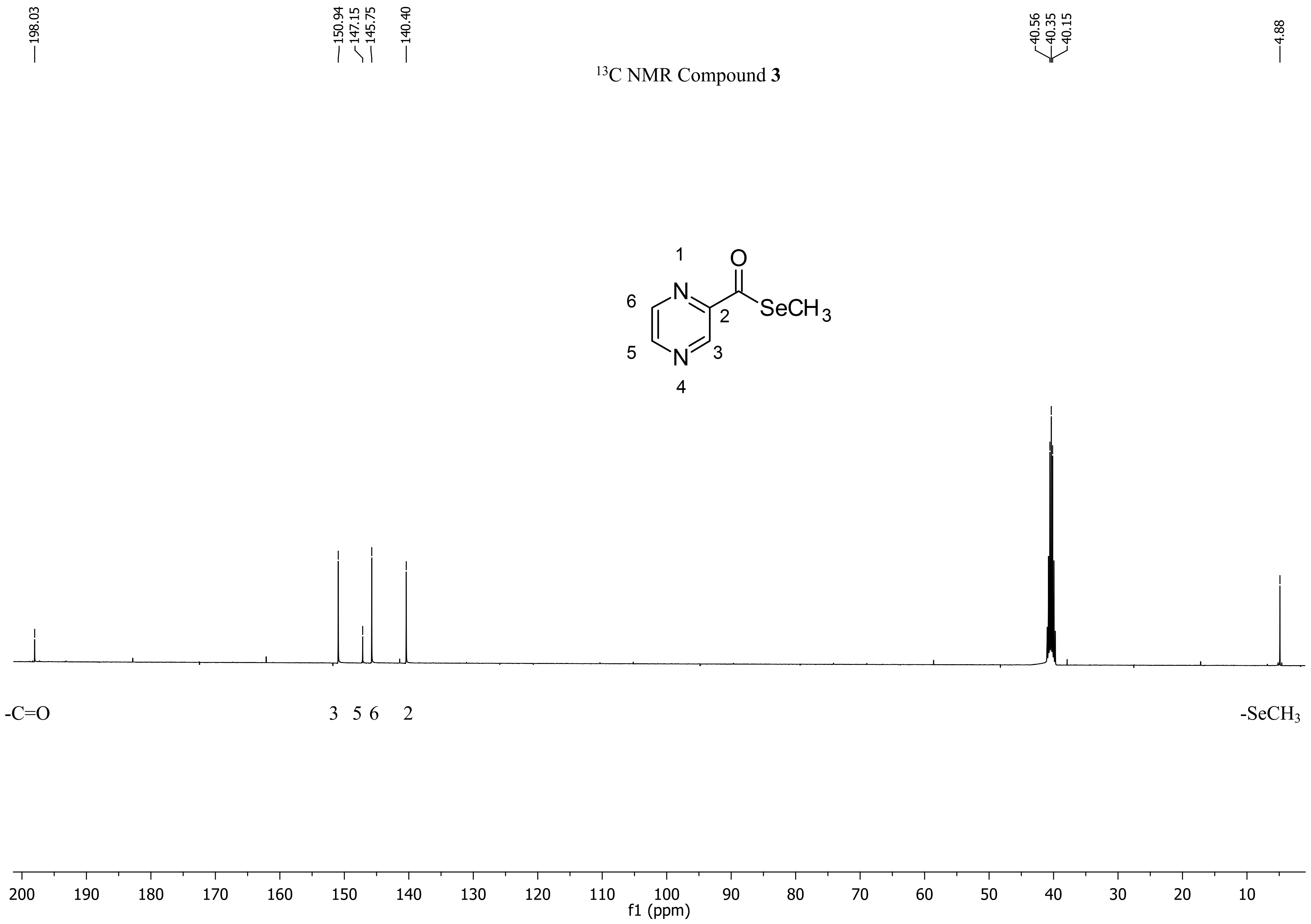

198.03
150.94
147.15
145.75
140.40
40.56
40.35
40.15
4.88
^{13}C NMR Compound **3**
1
6
5
4
2
3
O
N
N
SeCH3
-C=O
3 5 6 2
-SeCH3
200 190 180 170 160 150 140 130 120 110 100 90 80 70 60 50 40 30 20 10
f1 (ppm)

^{1}H NMR Compound **4**

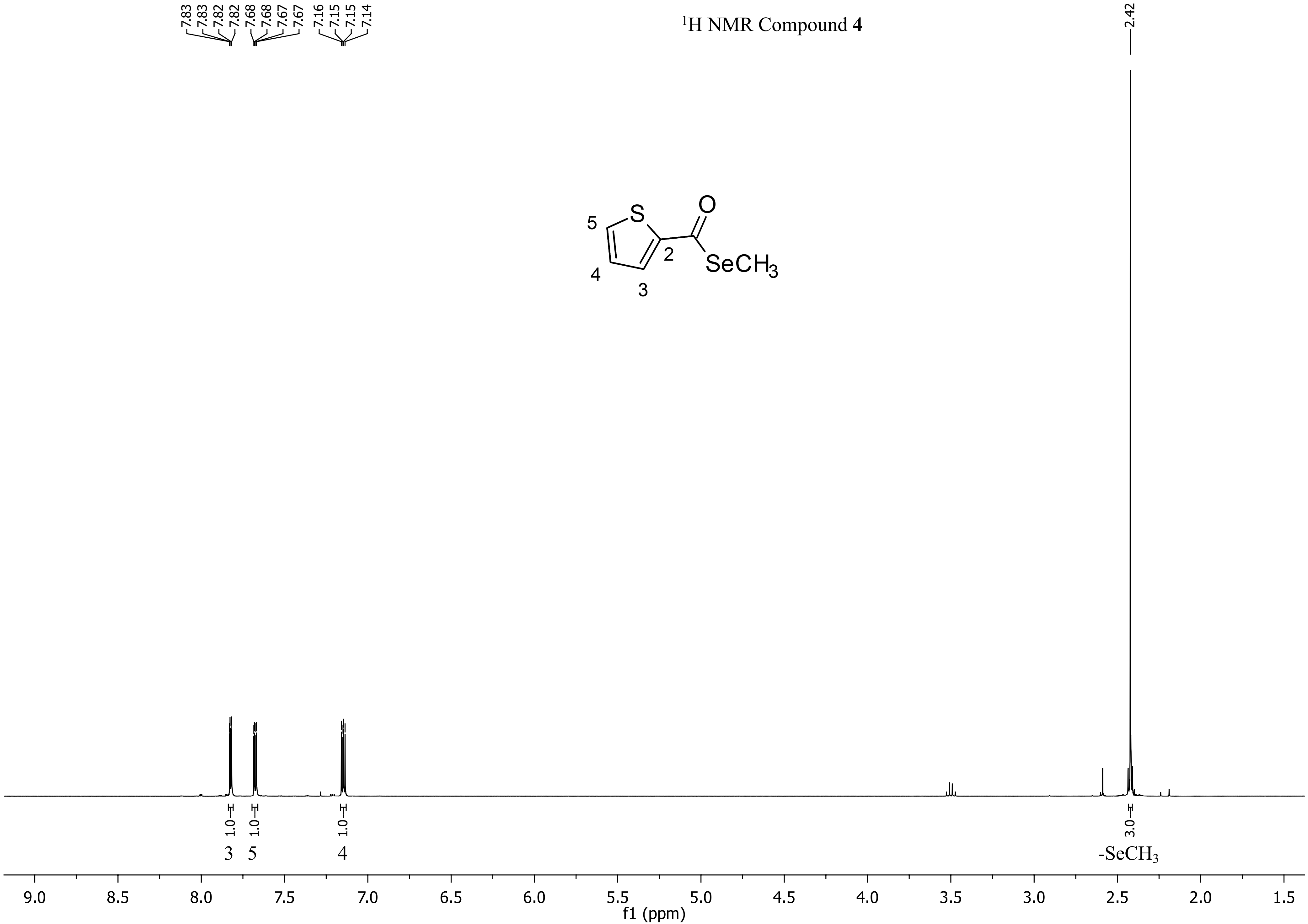

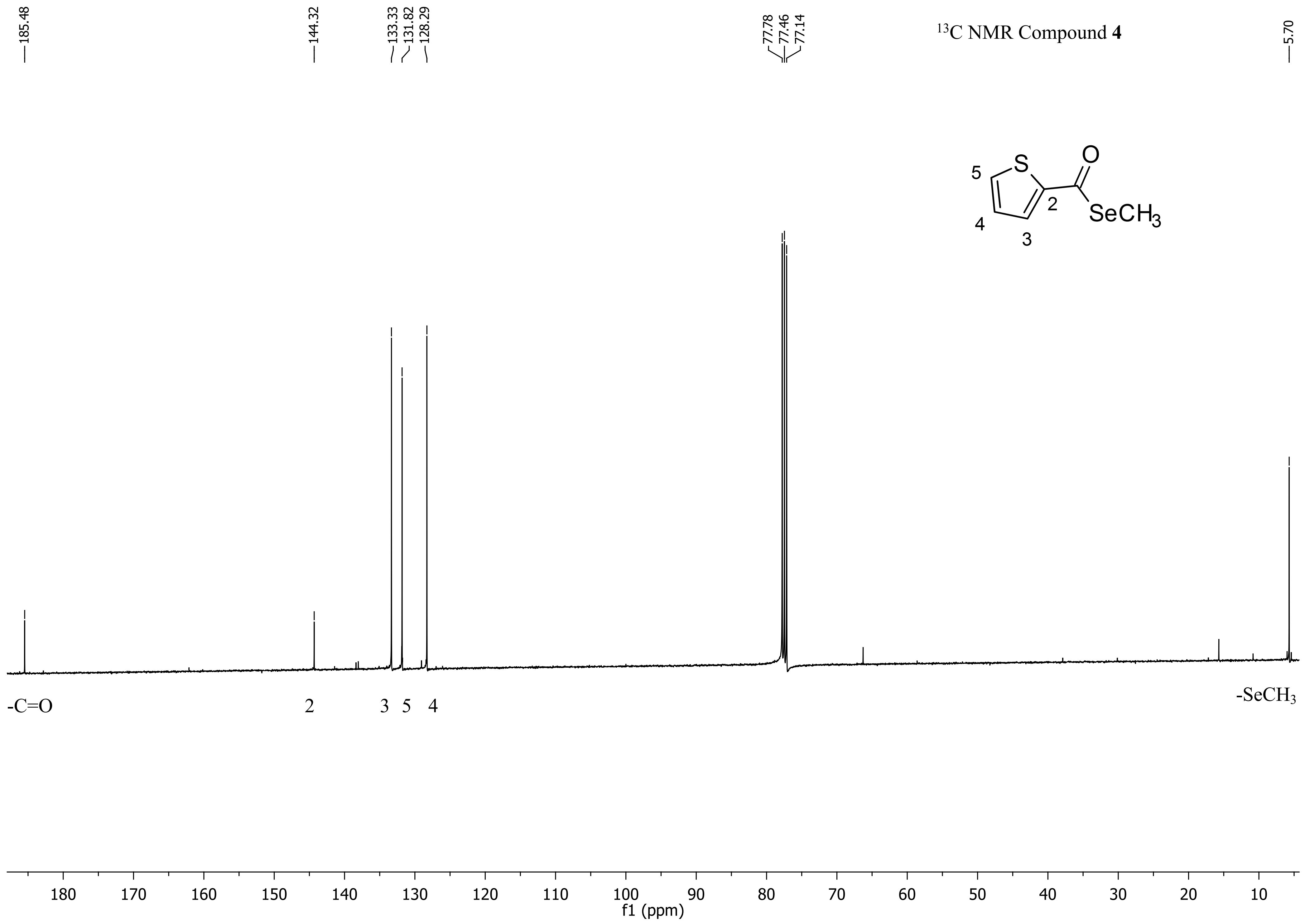

185.48
144.32
133.33
131.82
128.29
77.78
77.46
77.14
5.70
13C NMR Compound 4
S
O
SeCH3
5
4
3
2
-C=O
2
3 5 4
-SeCH3
180 170 160 150 140 130 120 110 100 90 80 70 60 50 40 30 20 10
f1 (ppm)

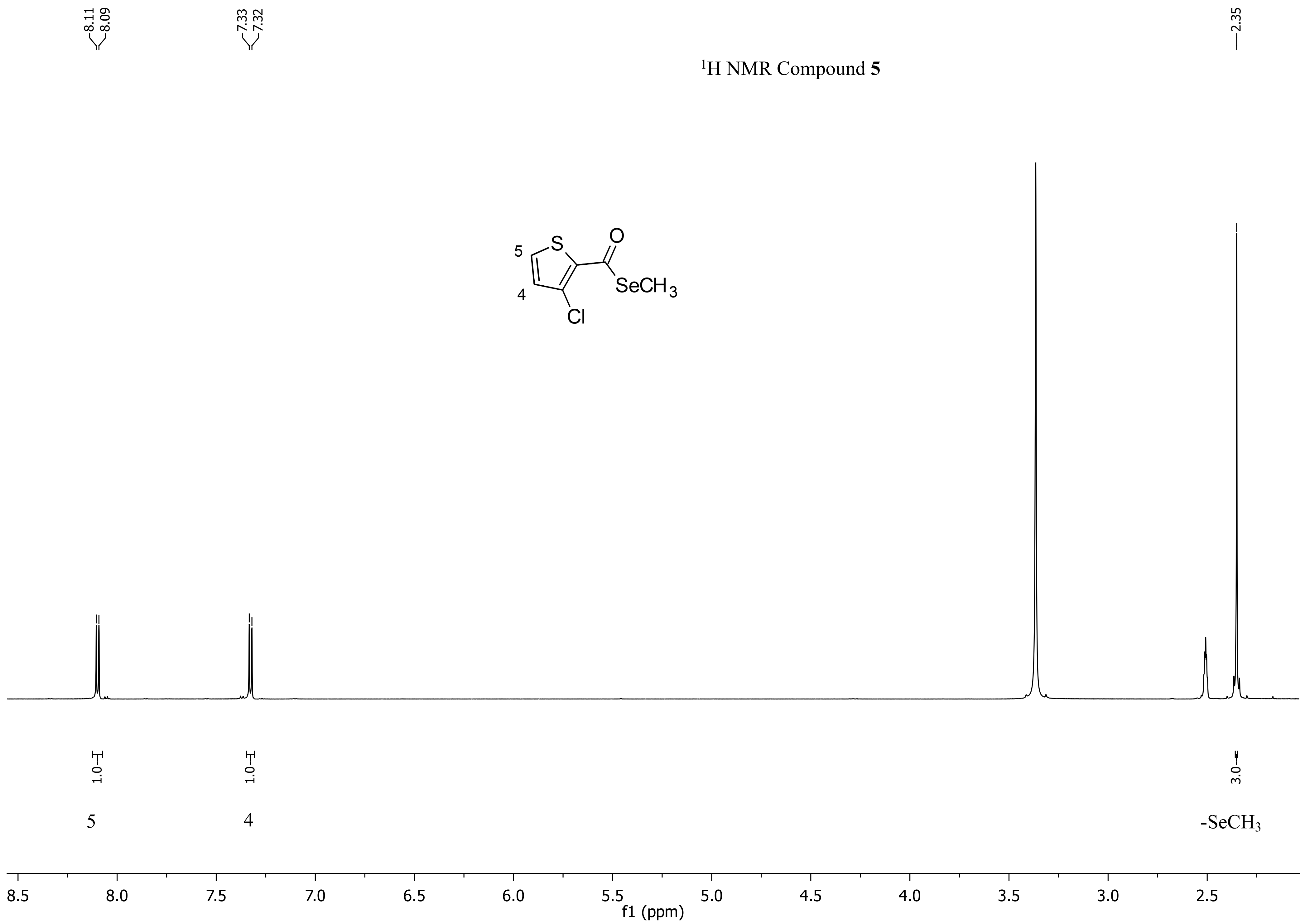

^{1}H NMR Compound **5**
8.11
8.09
7.33
7.32
2.35
5
4
S
O
SeCH3
Cl
1.0
1.0
3.0
5
4
-SeCH3
8.5
8.0
7.5
7.0
6.5
6.0
5.5
5.0
4.5
4.0
3.5
3.0
2.5
f1 (ppm)

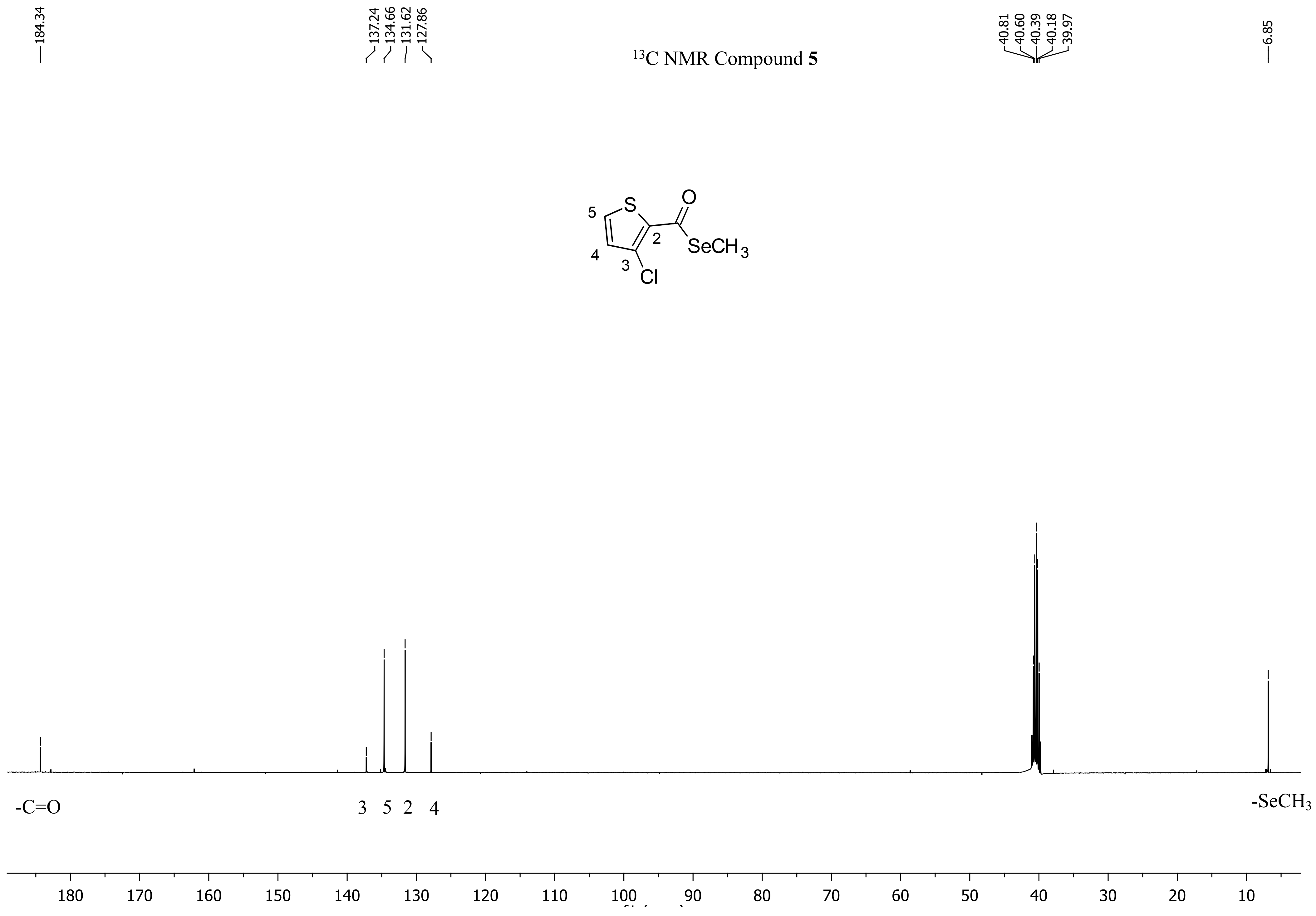
184.34
137.24
134.66
131.62
127.86
^{13}C NMR Compound **5**
40.81
40.60
40.39
40.18
39.97
6.85
S
O
5
2
SeCH3
4
3
Cl
-C=O
3 5 2 4
-SeCH3
180 170 160 150 140 130 120 110 100 90 80 70 60 50 40 30 20 10

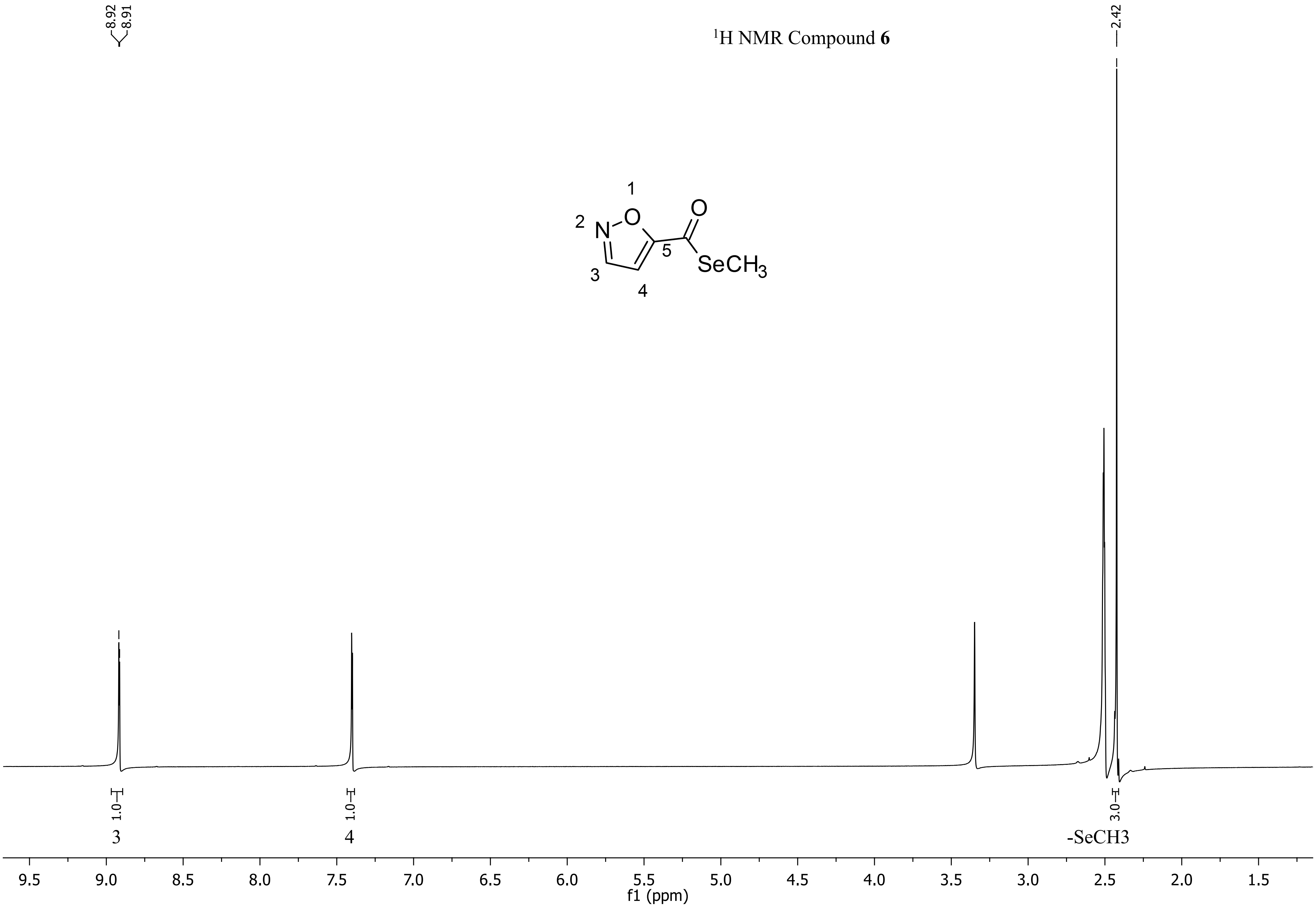
8.92
8.91
2.42
1H NMR Compound 6
1
2
N
O
O
5
SeCH3
3
4
1.0
1.0
3.0
3
4
-SeCH3
9.5
9.0
8.5
8.0
7.5
7.0
6.5
6.0
5.5
5.0
4.5
4.0
3.5
3.0
2.5
2.0
1.5
f1 (ppm)

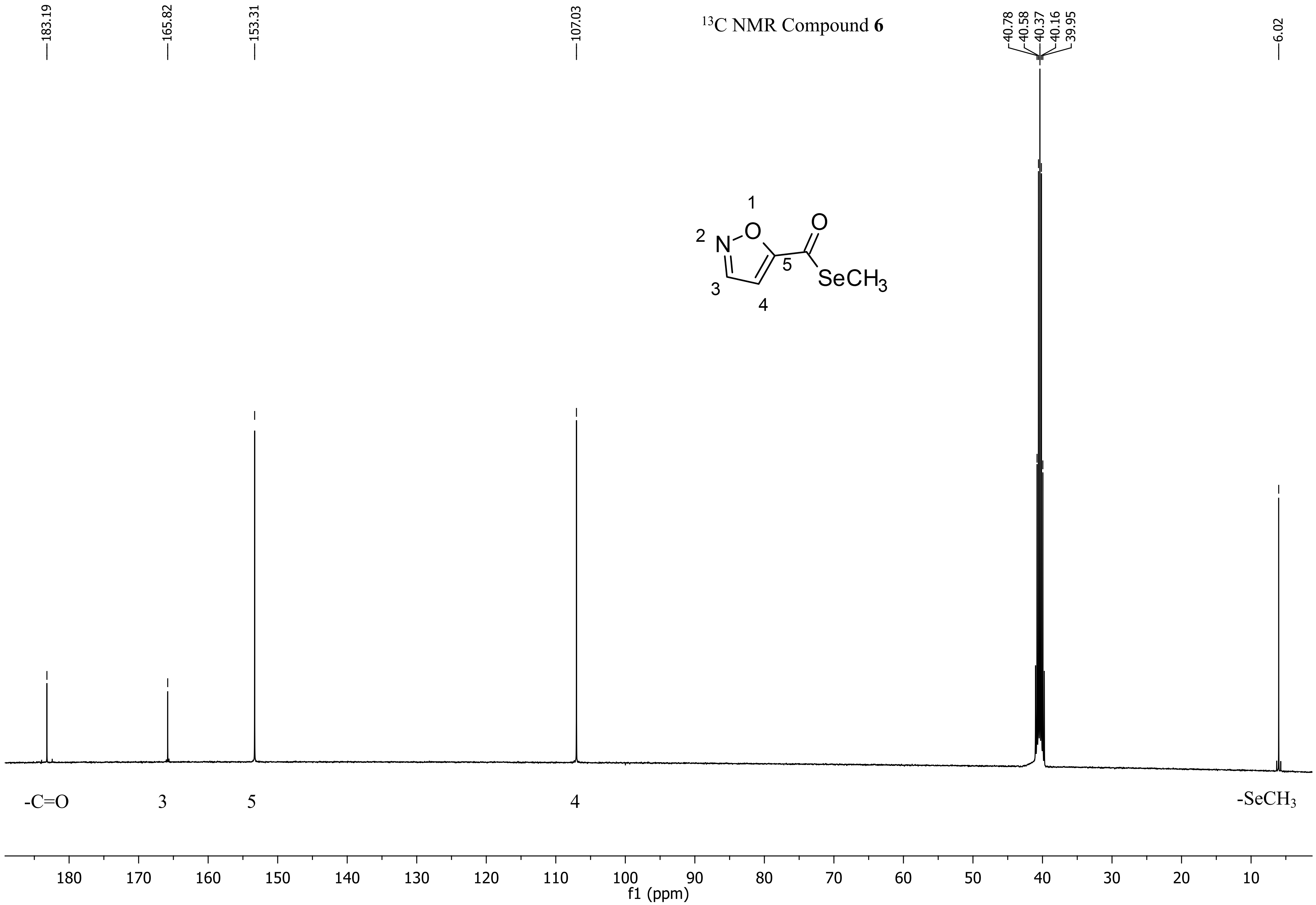

183.19
165.82
153.31
107.03
^{13}C NMR Compound **6**
40.78
40.58
40.37
40.16
39.95
6.02
1
2
3
4
5
O
N
SeCH$_3$
-C=O
3
5
4
-SeCH$_3$
180
170
160
150
140
130
120
110
100
90
80
70
60
50
40
30
20
10
f1 (ppm)

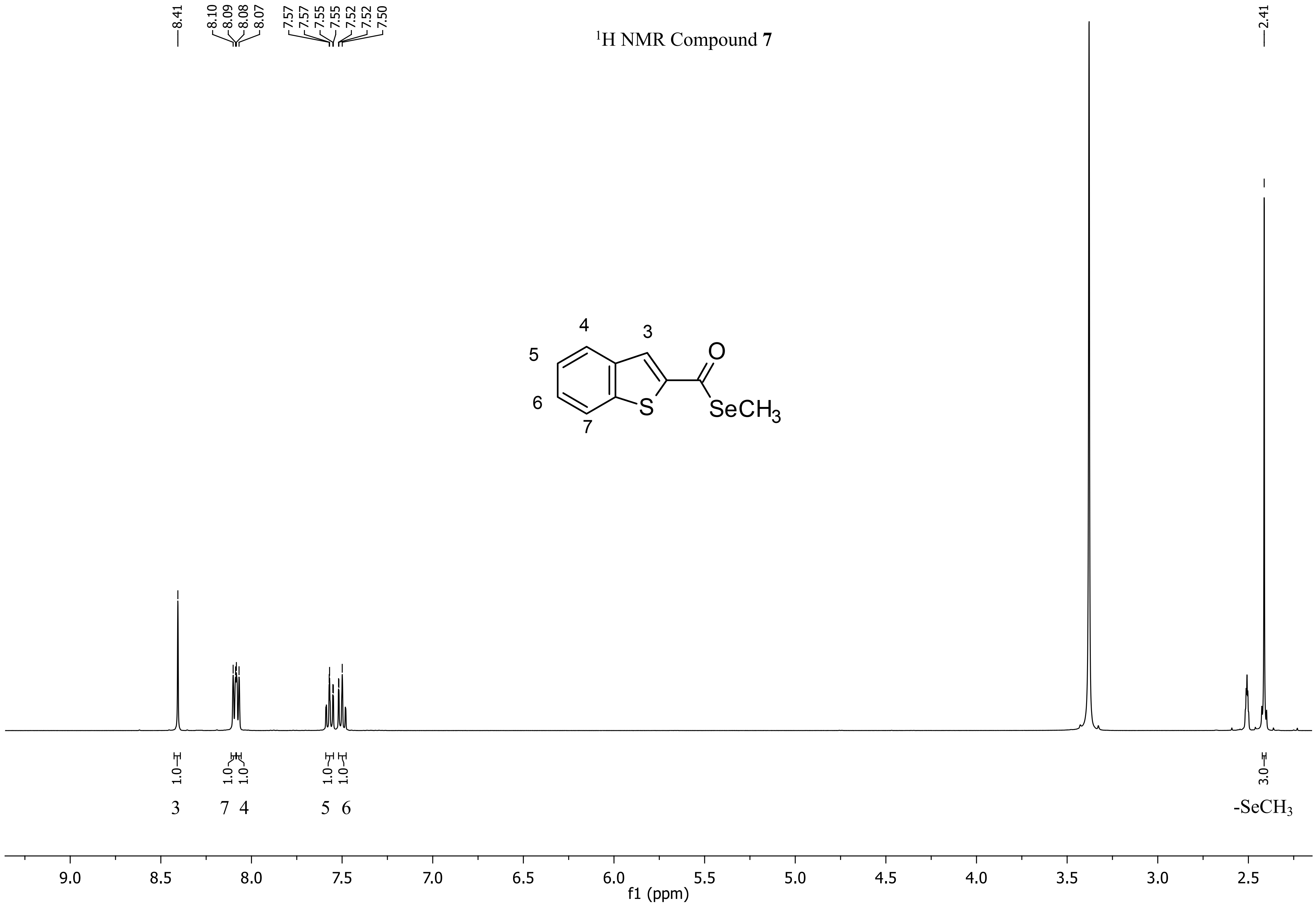

1H NMR Compound 7
8.41
8.10 8.09 8.08 8.07
7.57 7.57 7.55 7.55 7.52 7.52 7.50
2.41
4
3
5
6
7
O
S
SeCH3
1.0
1.0
1.0
1.0
1.0
3.0
3 7 4 5 6 -SeCH3
9.0 8.5 8.0 7.5 7.0 6.5 6.0 5.5 5.0 4.5 4.0 3.5 3.0 2.5
f1 (ppm)

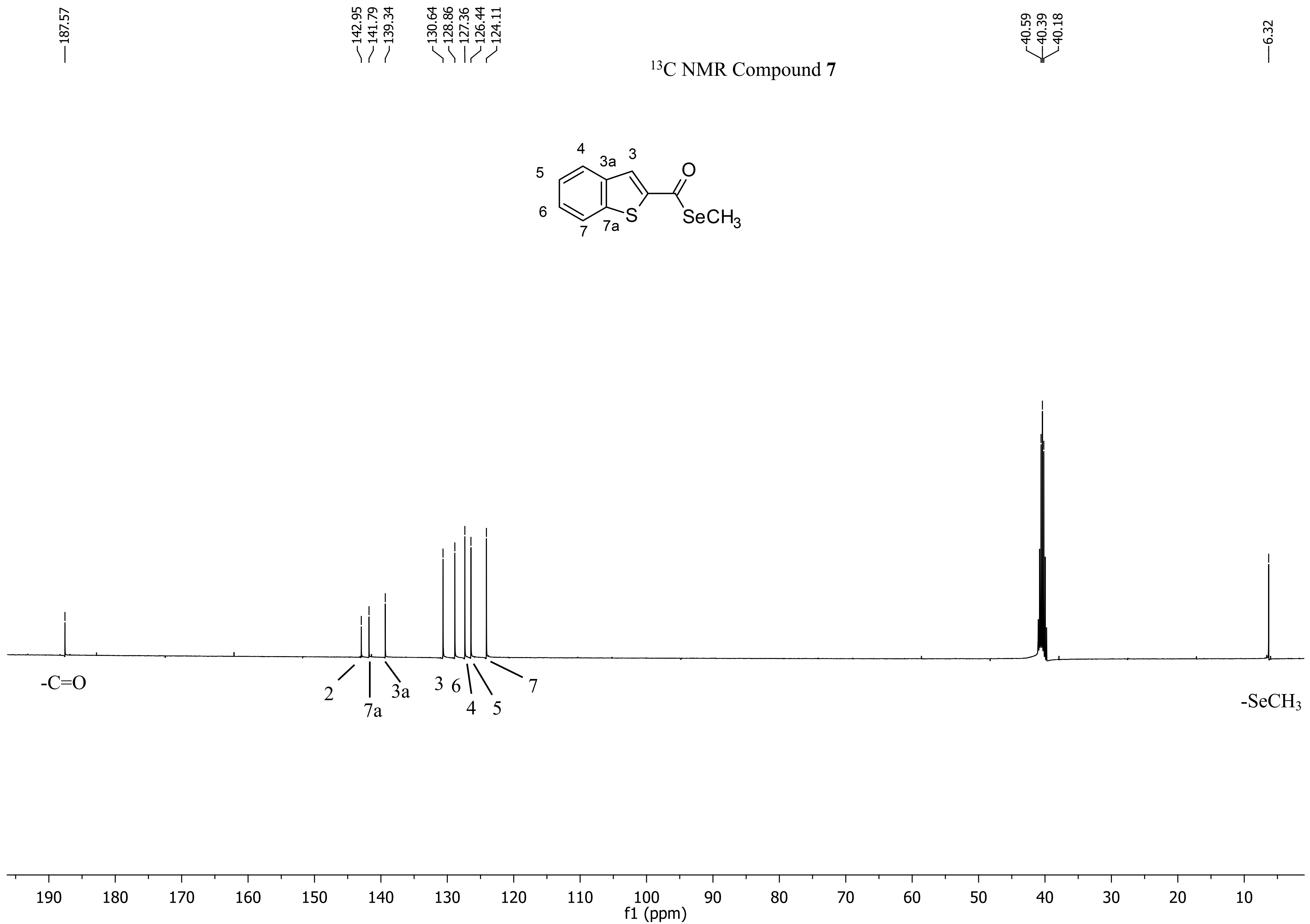

^{13}C NMR Compound **7**
187.57
142.95
141.79
139.34
130.64
128.86
127.36
126.44
124.11
40.59
40.39
40.18
6.32
4
3a
3
5
6
7
7a
S
O
SeCH3
-C=O
2
7a
3a
3
6
4
5
7
-SeCH3
190
180
170
160
150
140
130
120
110
100
90
80
70
60
50
40
30
20
10
f1 (ppm)

^{1}H NMR Compound **8**

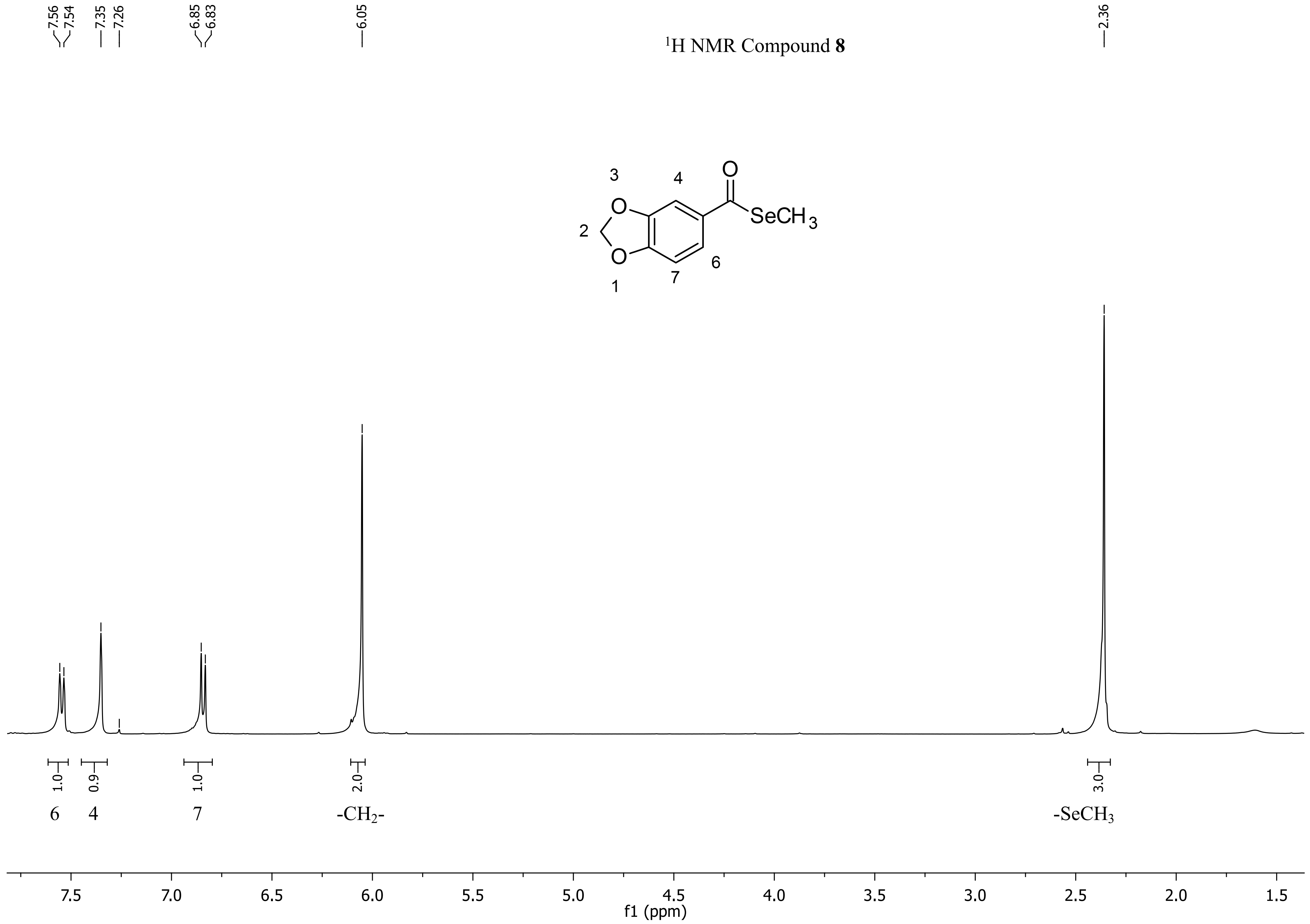

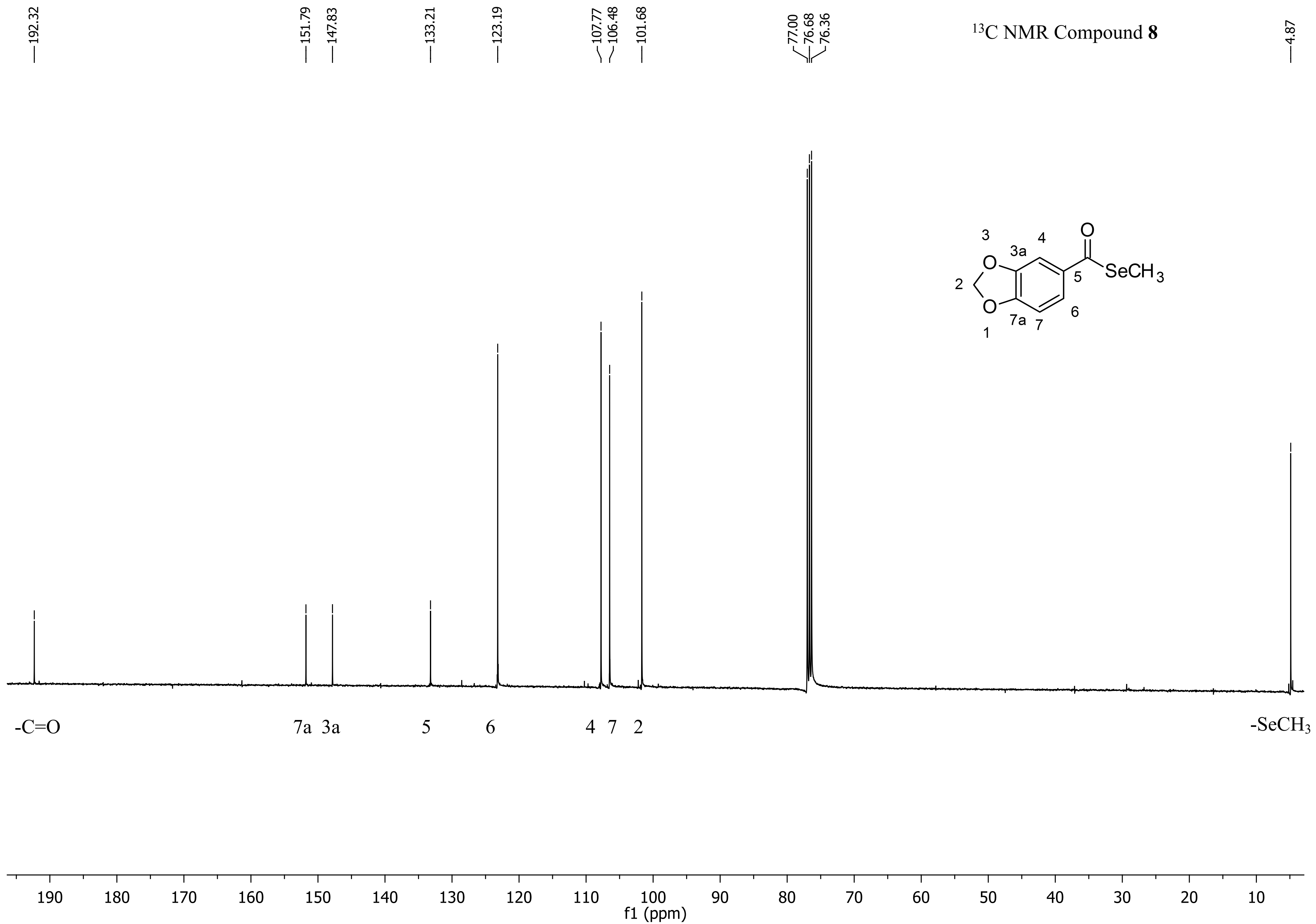

192.32
151.79
147.83
133.21
123.19
107.77
106.48
101.68
77.00
76.68
76.36
^{13}C NMR Compound **8**
4.87
3
4
3a
O
2
5
SeCH$_3$
7a
7
6
1
-C=O
7a 3a
5
6
4 7 2
-SeCH$_3$
190 180 170 160 150 140 130 120 110 100 90 80 70 60 50 40 30 20 10
f1 (ppm)

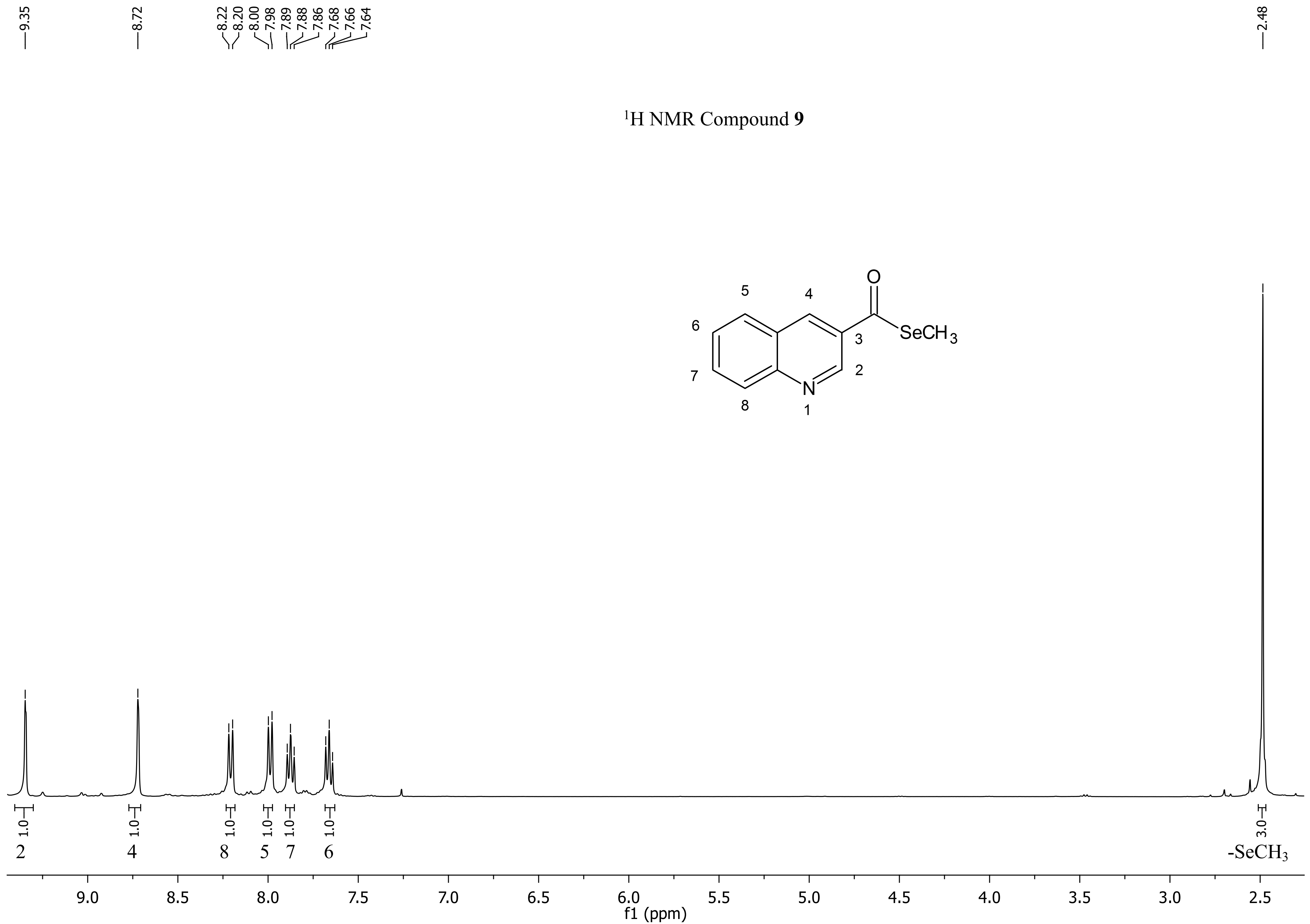

^{1}H NMR Compound **9**
9.35
8.72
8.22 8.20 8.00 7.98 7.89 7.88 7.86 7.68 7.66 7.64
2.48
O
SeCH$_3$
5 4 6 3 7 2 8 N 1
1.0 1.0 1.0 1.0 1.0 1.0 3.0
2 4 8 5 7 6 -SeCH$_3$
9.0 8.5 8.0 7.5 7.0 6.5 6.0 5.5 5.0 4.5 4.0 3.5 3.0 2.5
f1 (ppm)

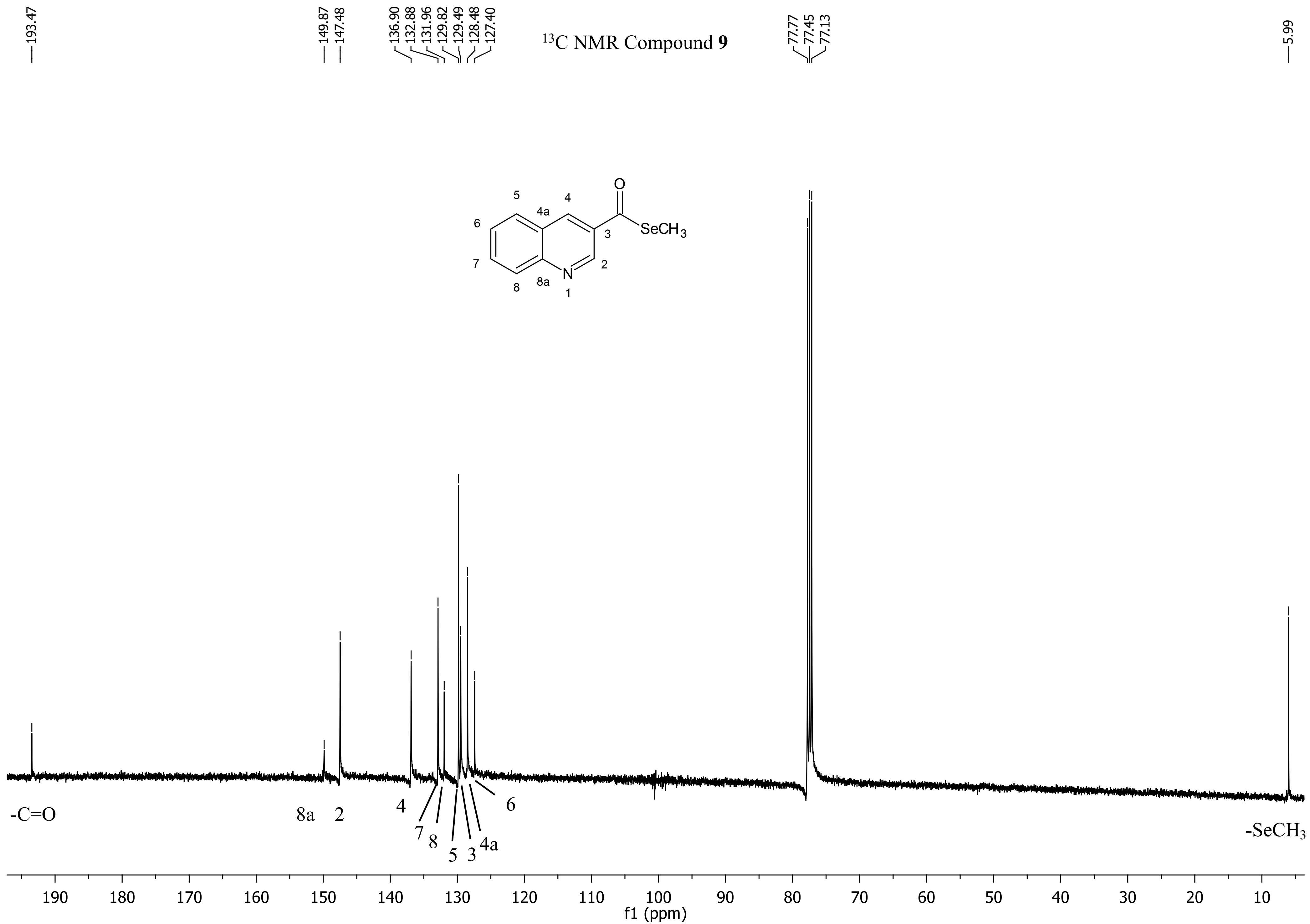

193.47
149.87
147.48
136.90
132.88
131.96
129.82
129.49
128.48
127.40
^{13}C NMR Compound **9**
77.77
77.45
77.13
5.99
O
SeCH3
5
4
4a
6
3
7
2
8
8a
N
1
-C=O
8a
2
4
7
8
5
3
4a
6
-SeCH3
190
180
170
160
150
140
130
120
110
100
90
80
70
60
50
40
30
20
10
f1 (ppm)

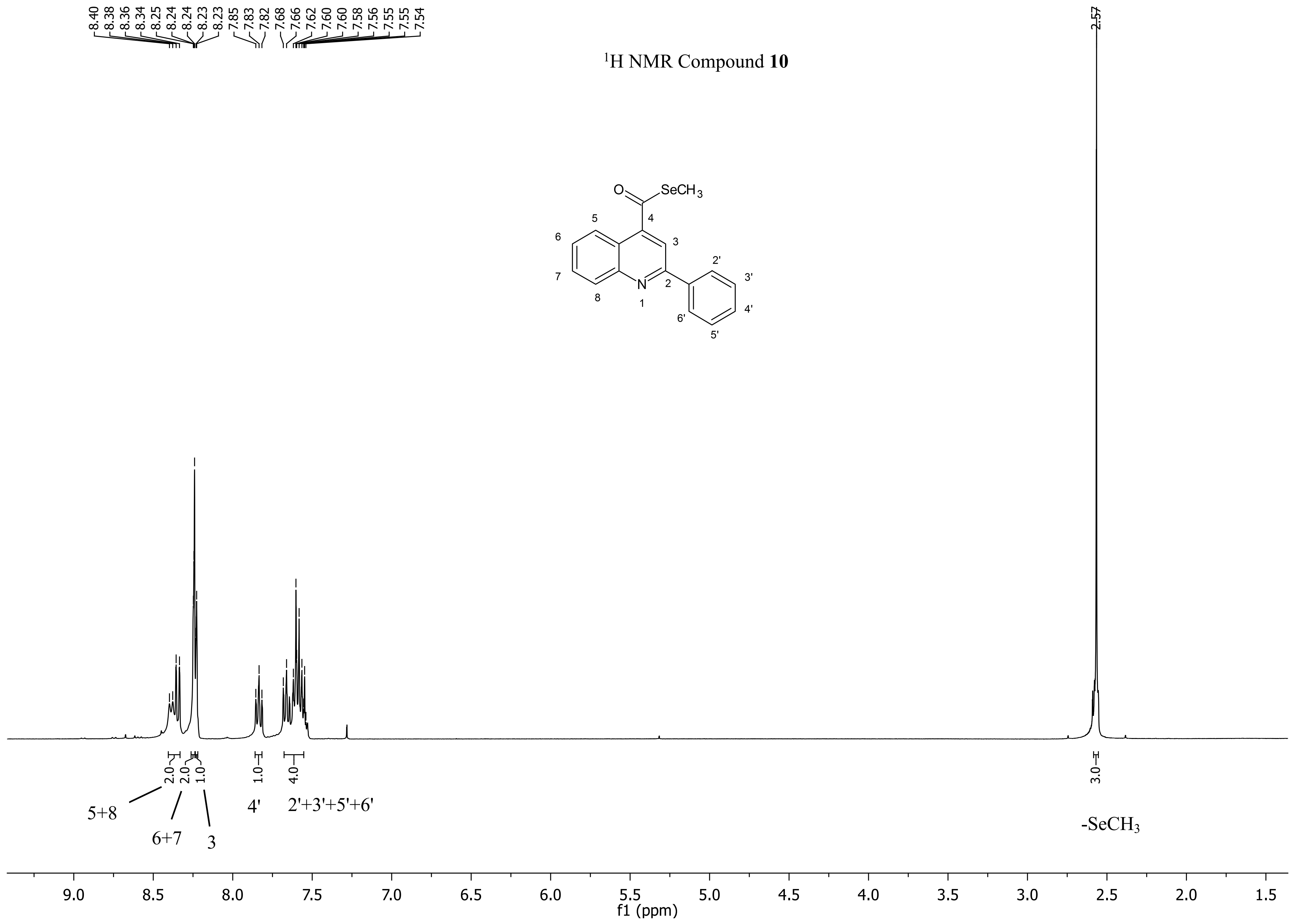
8.40
8.38
8.36
8.34
8.25
8.24
8.24
8.23
8.23
7.85
7.83
7.82
7.68
7.66
7.62
7.60
7.60
7.58
7.56
7.55
7.55
7.54
2.57
1H NMR Compound 10
O
SeCH3
5
4
6
3
2'
7
N
2
3'
8
1
4'
6'
5'
2.0
2.0
1.0
1.0
4.0
3.0
5+8
6+7
3
4'
2'+3'+5'+6'
-SeCH3
9.0
8.5
8.0
7.5
7.0
6.5
6.0
5.5
5.0
4.5
4.0
3.5
3.0
2.5
2.0
1.5
f1 (ppm)

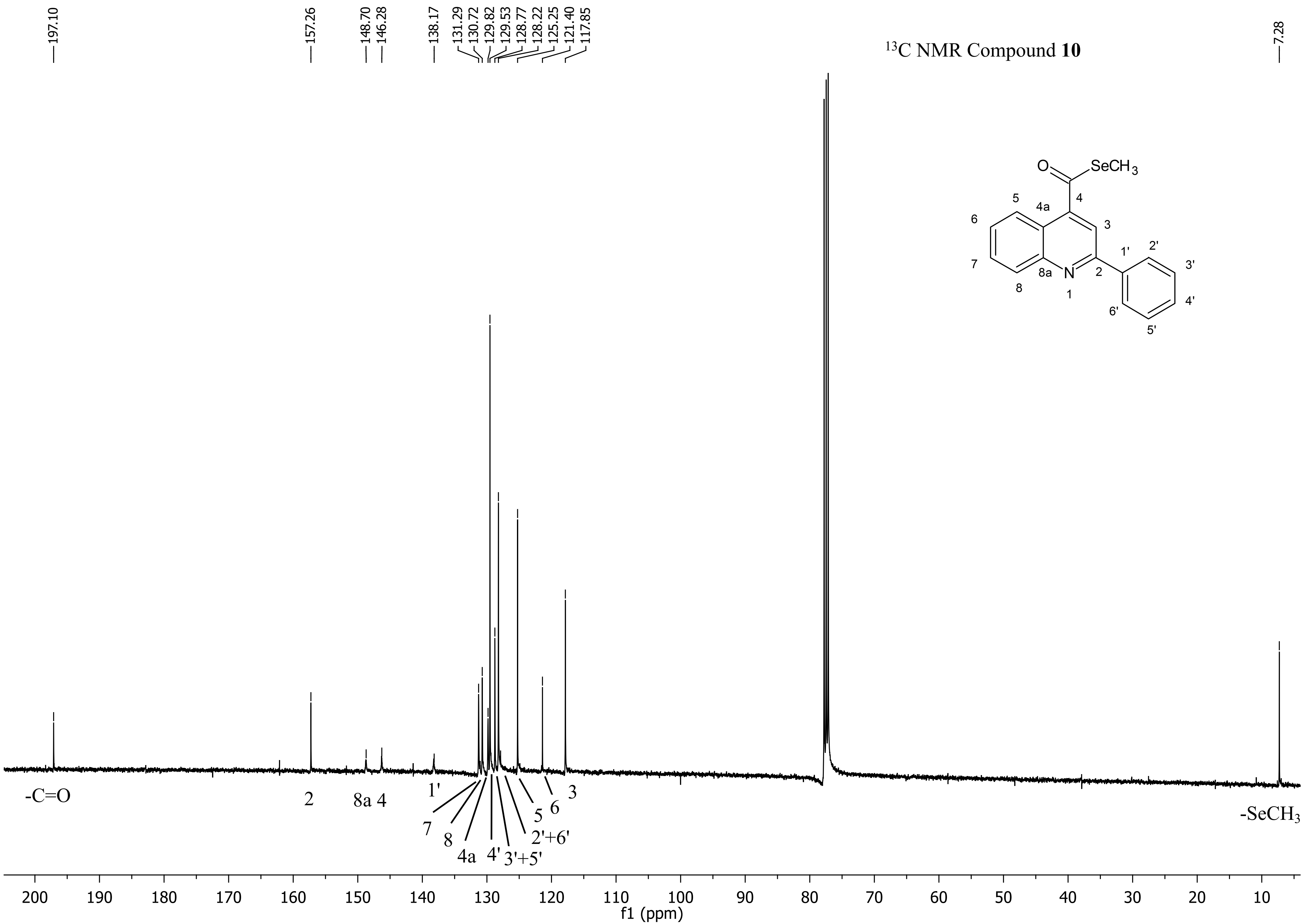

197.10
157.26
148.70
146.28
138.17
131.29
130.72
129.82
129.53
128.77
128.22
125.25
121.40
117.85
7.28
13C NMR Compound 10
SeCH3
O
-C=O
2
8a 4
1'
7
8
4a
4'
3'+5'
2'+6'
5
6
3
-SeCH3
f1 (ppm)

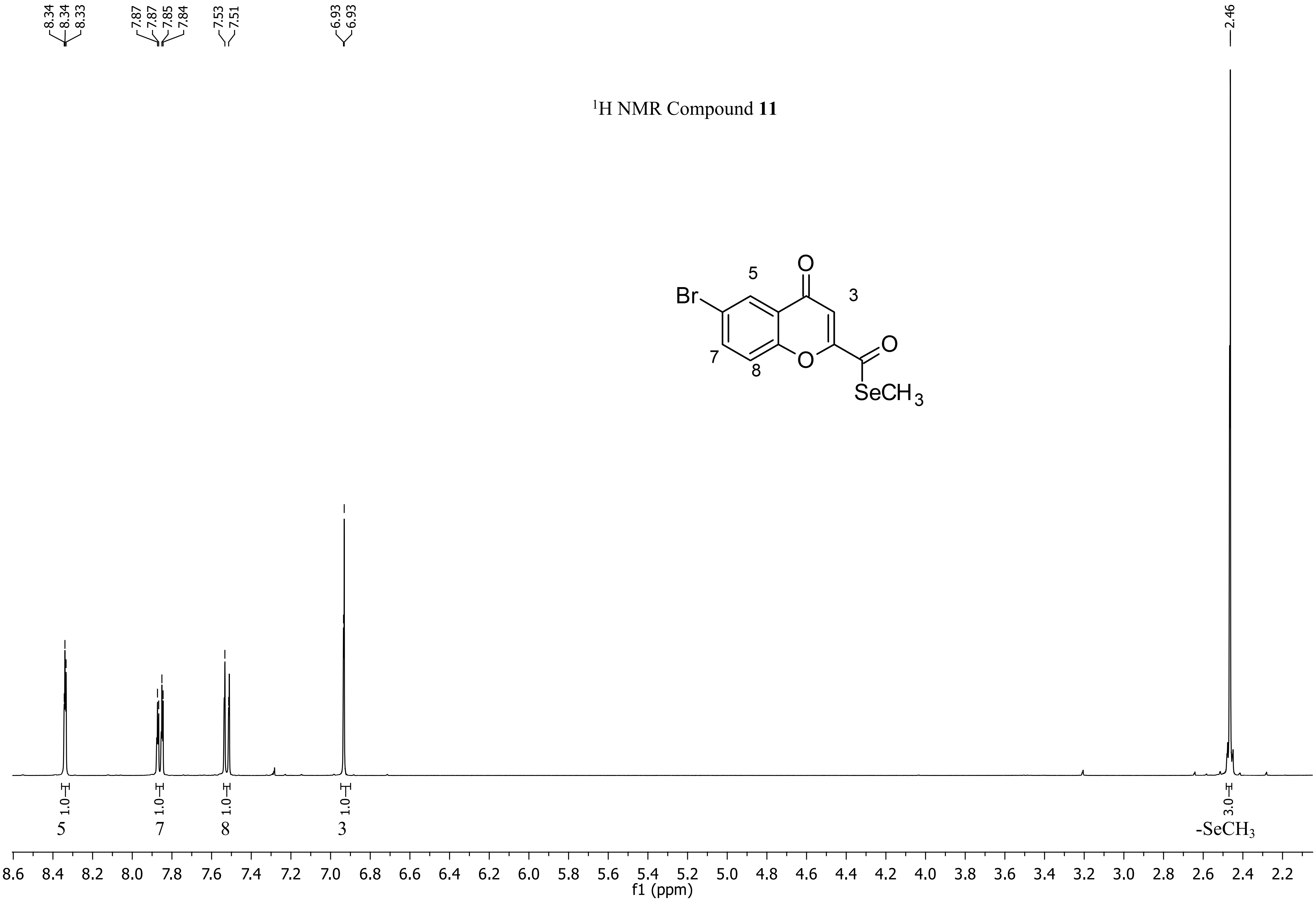

8.34
8.34
8.33
7.87
7.87
7.85
7.84
7.53
7.51
6.93
6.93
2.46
¹H NMR Compound **11**
Br
O
5
3
7
8
O
SeCH3
1.0
1.0
1.0
1.0
3.0
5
7
8
3
-SeCH3
8.6 8.4 8.2 8.0 7.8 7.6 7.4 7.2 7.0 6.8 6.6 6.4 6.2 6.0 5.8 5.6 5.4 5.2 5.0 4.8 4.6 4.4 4.2 4.0 3.8 3.6 3.4 3.2 3.0 2.8 2.6 2.4 2.2
f1 (ppm)

^{13}C NMR Compound **11**

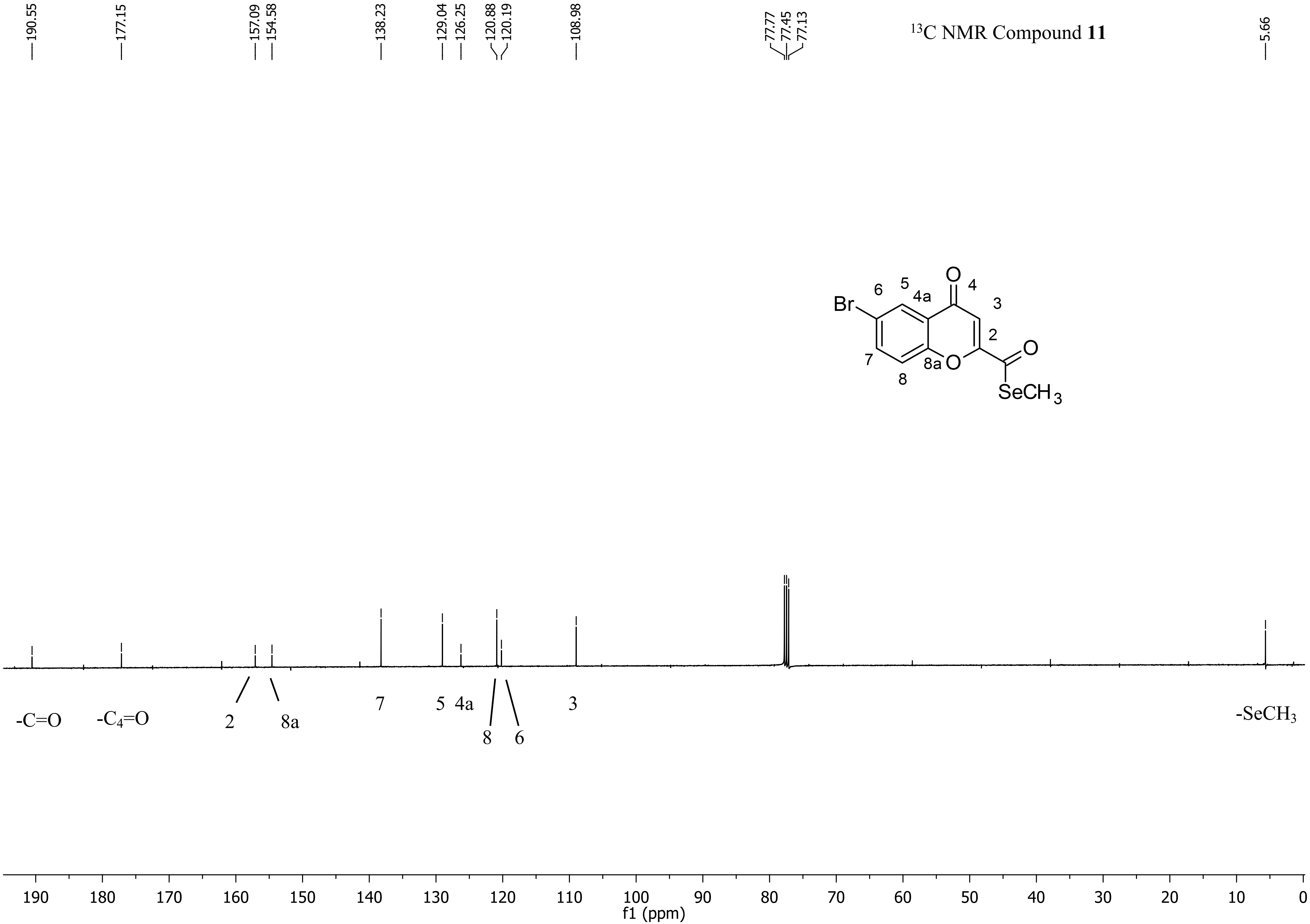

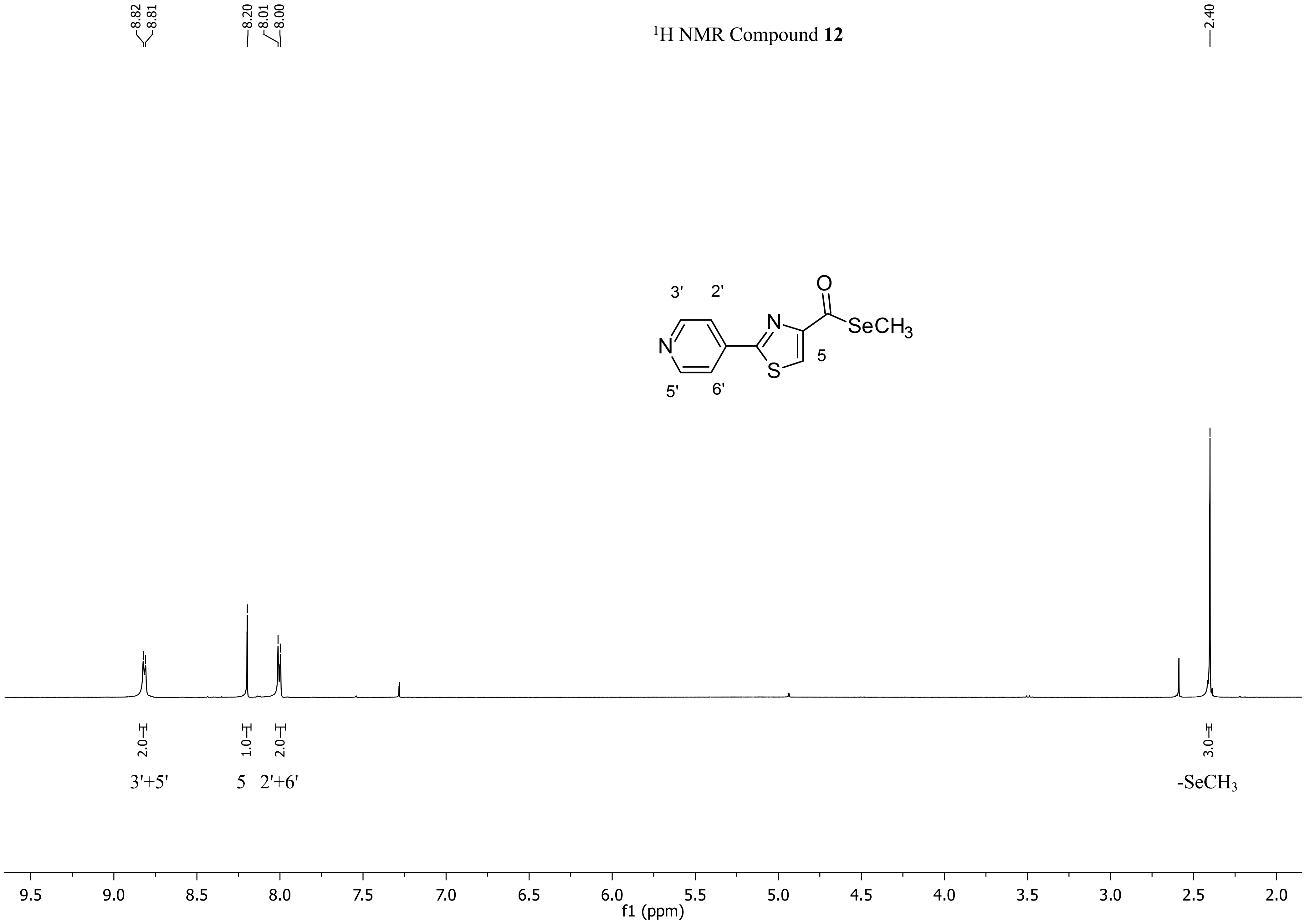

8.82
8.81
8.20
8.01
8.00
2.40
¹H NMR Compound 12
3'
2'
O
N
SeCH3
N
5
S
5'
6'
2.0
1.0
2.0
3.0
3'+5'
5
2'+6'
-SeCH3
9.5
9.0
8.5
8.0
7.5
7.0
6.5
6.0
5.5
5.0
4.5
4.0
3.5
3.0
2.5
2.0
f1 (ppm)

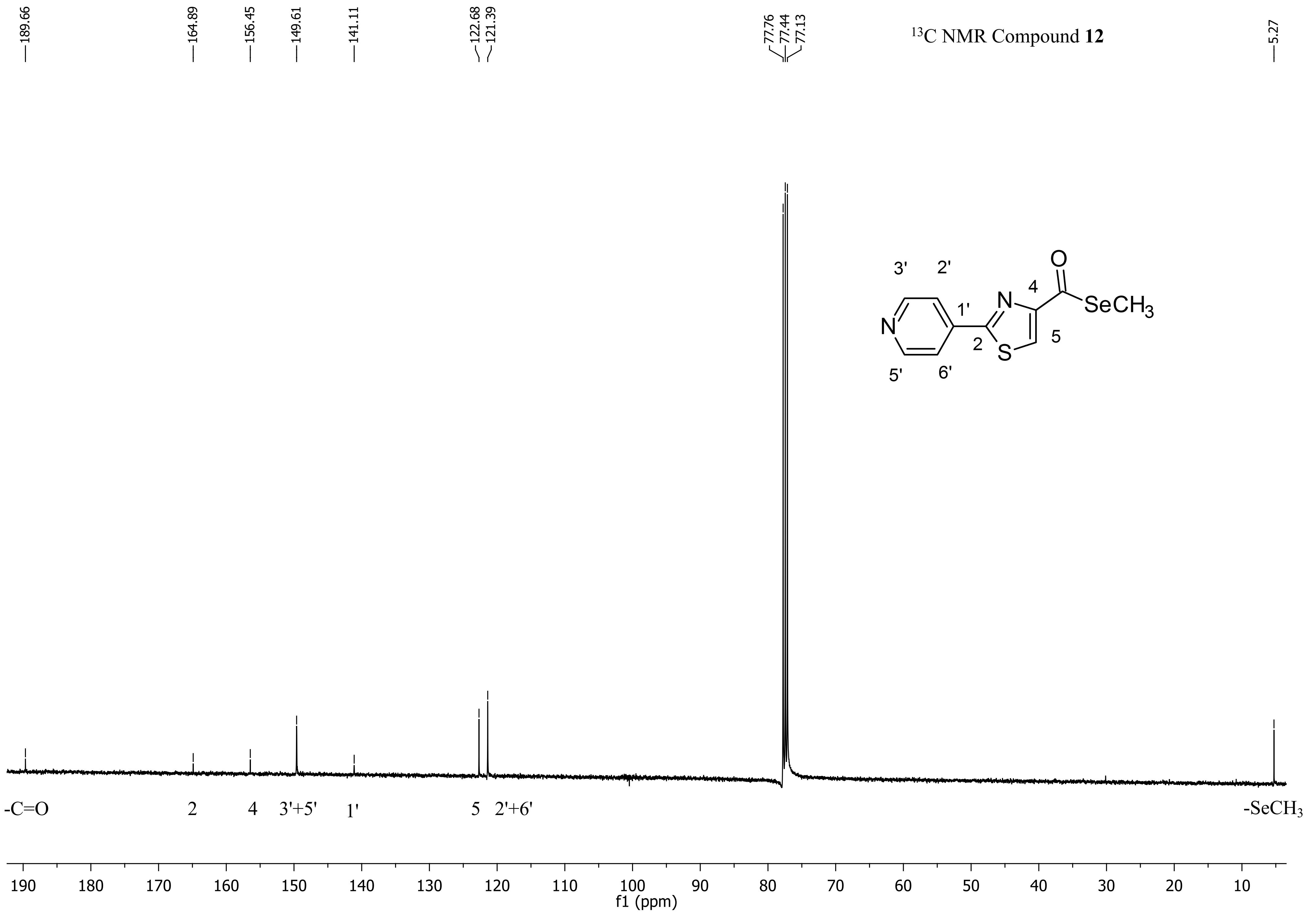

13C NMR Compound 12
189.66
164.89
156.45
149.61
141.11
122.68
121.39
77.76
77.44
77.13
5.27
3'
2'
1'
N
S
4
2
5
5'
6'
O
SeCH3
-C=O
2
4
3'+5'
1'
5
2'+6'
-SeCH3
190
180
170
160
150
140
130
120
110
100
90
80
70
60
50
40
30
20
10
f1 (ppm)

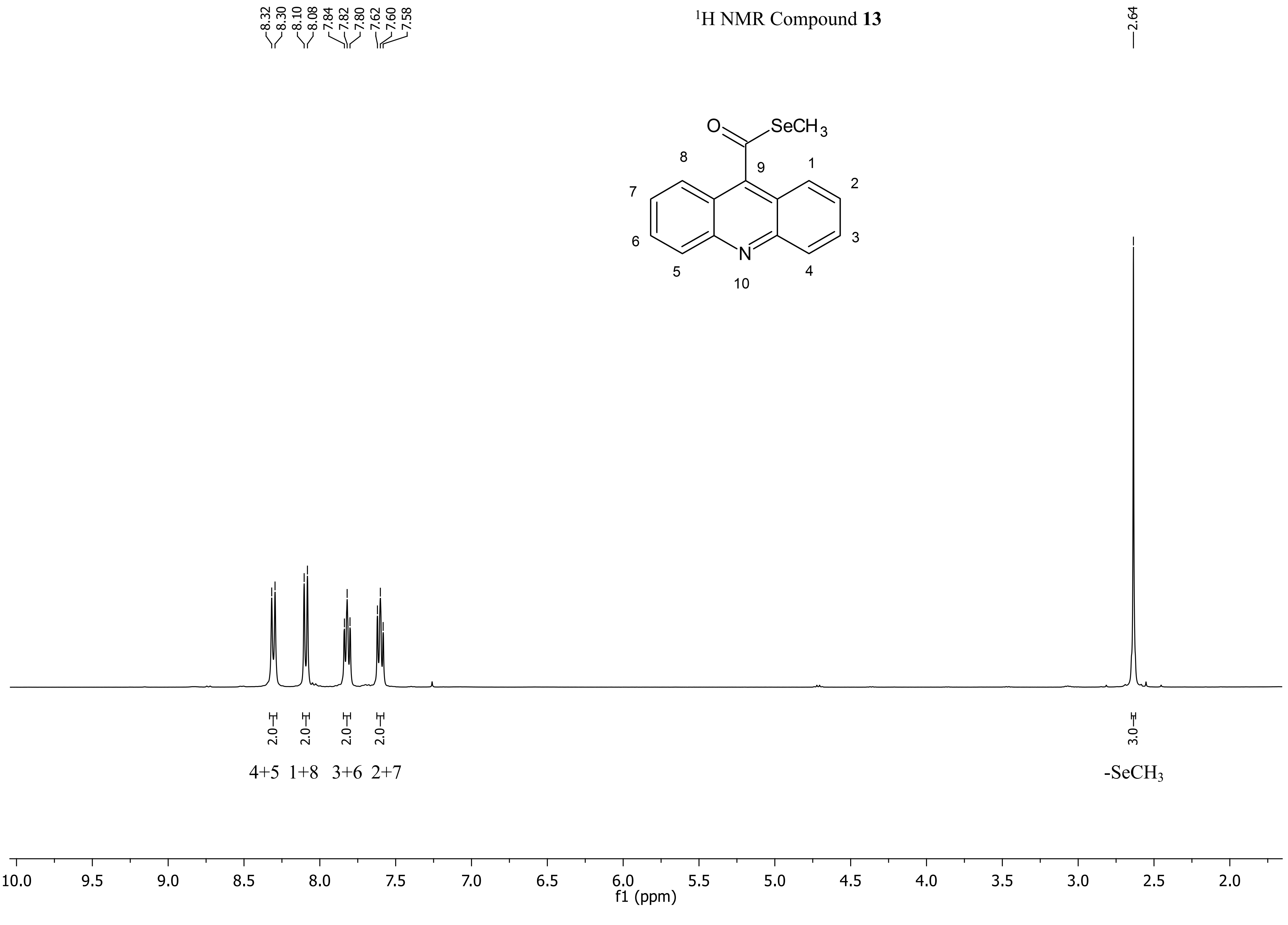

^{1}H NMR Compound **13**
8.32
8.30
8.10
8.08
7.84
7.82
7.80
7.62
7.60
7.58
2.64
O
SeCH3
1
2
3
4
5
6
7
8
9
10
N
2.0
2.0
2.0
2.0
3.0
4+5
1+8
3+6
2+7
-SeCH3
10.0
9.5
9.0
8.5
8.0
7.5
7.0
6.5
6.0
5.5
5.0
4.5
4.0
3.5
3.0
2.5
2.0
f1 (ppm)

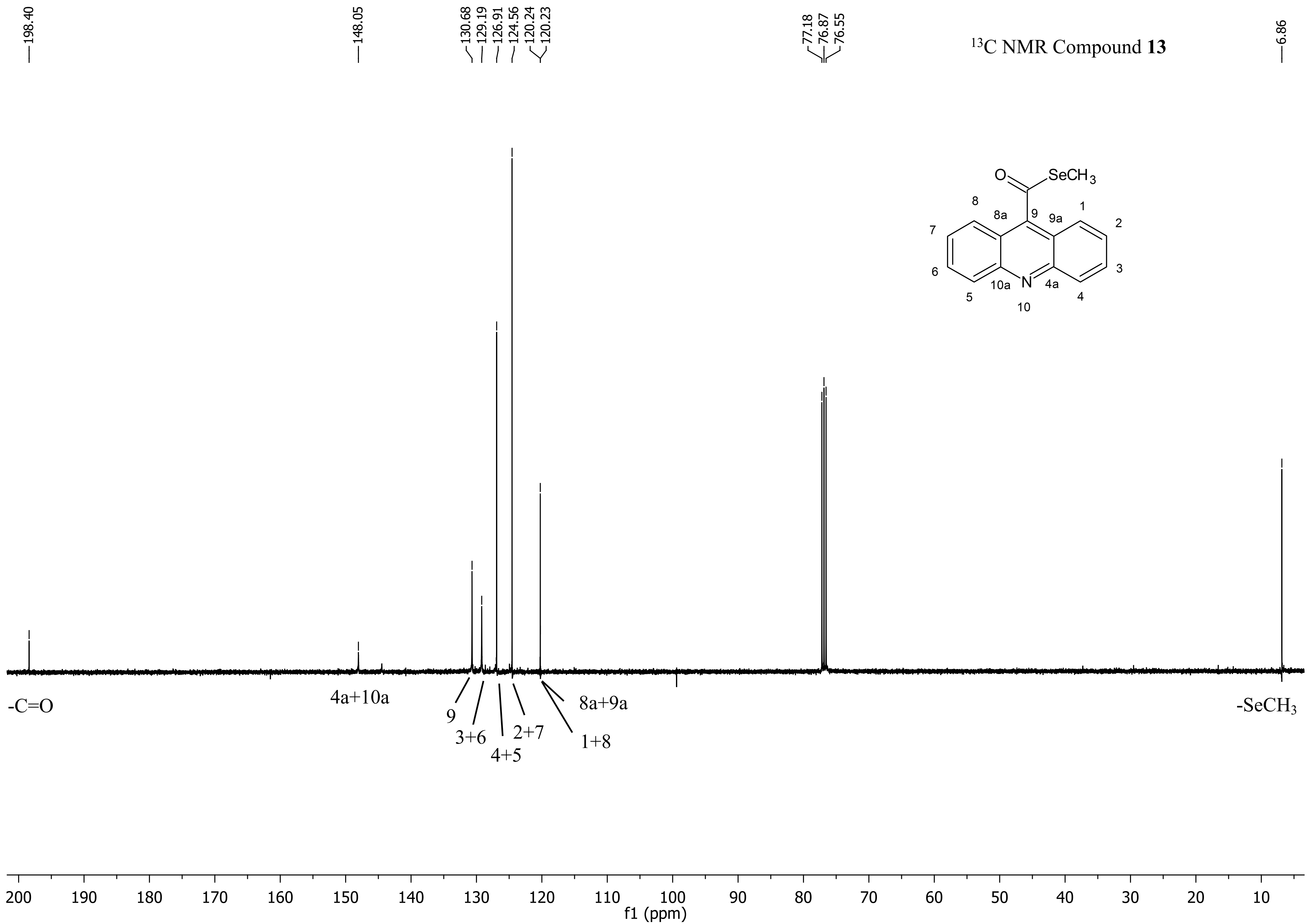

198.40
148.05
130.68
129.19
126.91
124.56
120.24
120.23
77.18
76.87
76.55
6.86
^{13}C NMR Compound **13**
O
SeCH$_3$
8
8a
9
9a
1
7
2
6
3
10a
N
4a
5
10
4
-C=O
4a+10a
9
3+6
4+5
2+7
8a+9a
1+8
-SeCH$_3$
200 190 180 170 160 150 140 130 120 110 100 90 80 70 60 50 40 30 20 10
f1 (ppm)

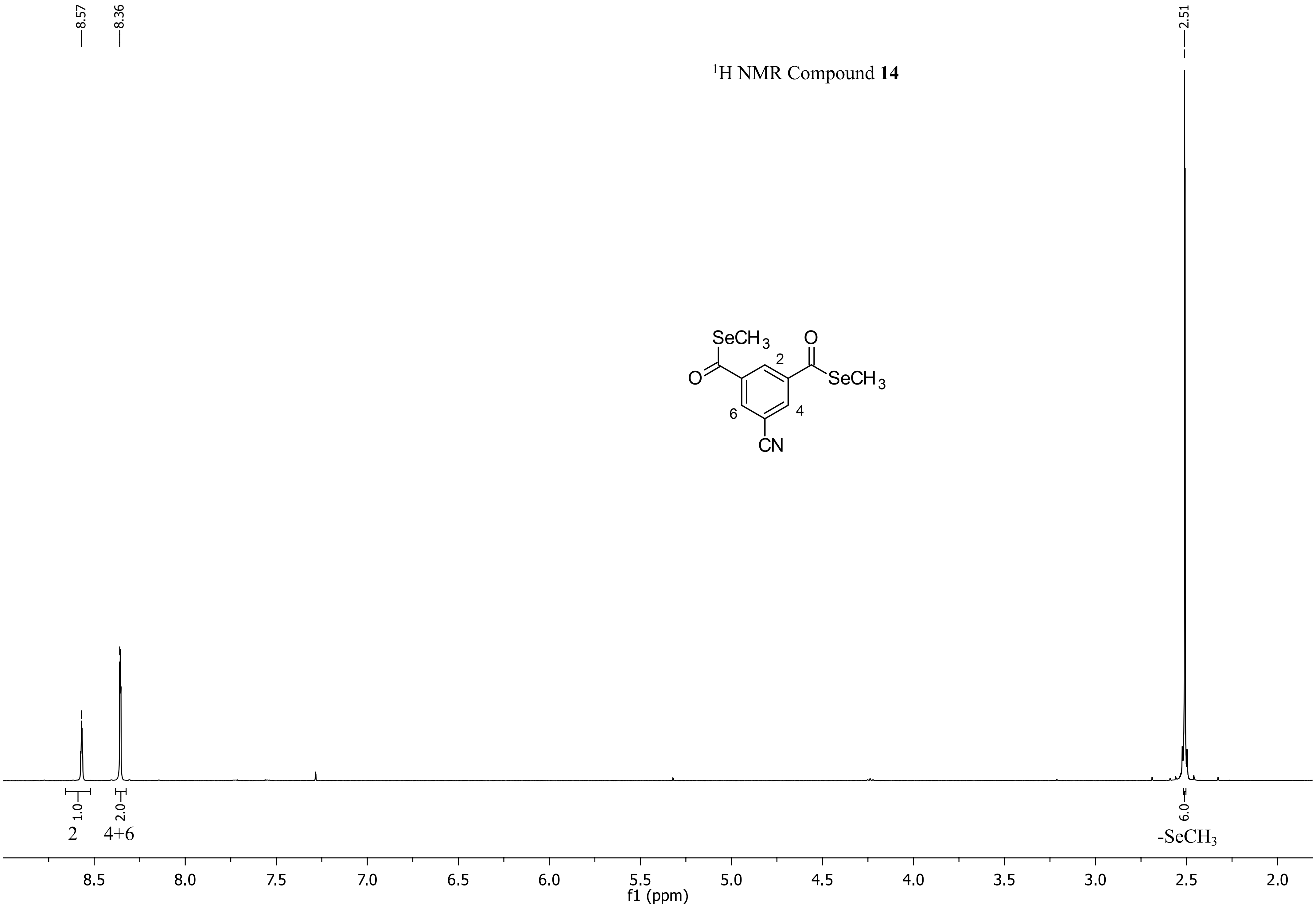

8.57
8.36
2.51
¹H NMR Compound **14**
SeCH3
O
2
O
SeCH3
6
4
CN
1.0
2.0
6.0
2
4+6
-SeCH3
8.5
8.0
7.5
7.0
6.5
6.0
5.5
5.0
4.5
4.0
3.5
3.0
2.5
2.0
f1 (ppm)

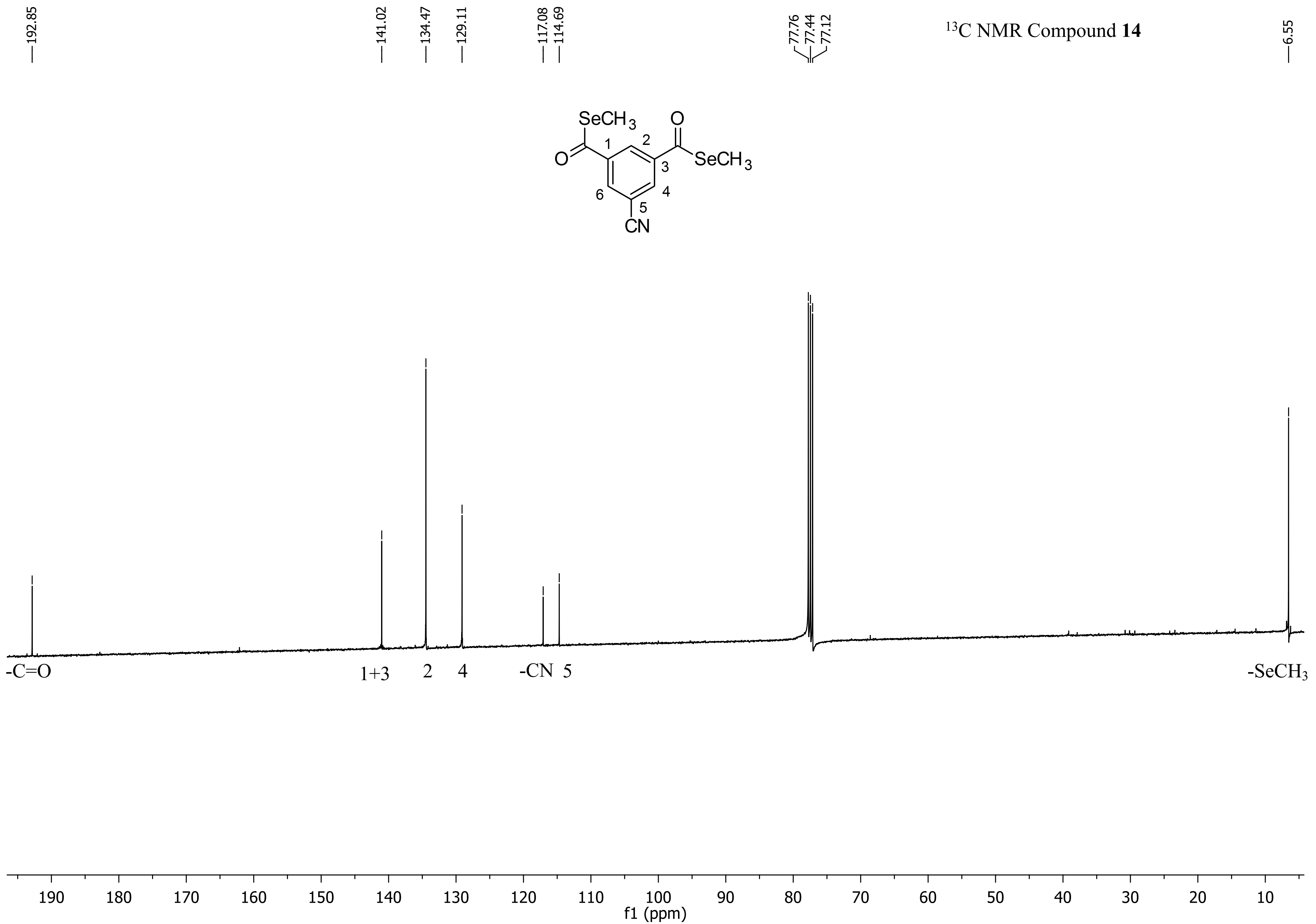

192.85
141.02
134.47
129.11
117.08
114.69
77.76
77.44
77.12
^{13}C NMR Compound **14**
6.55
$SeCH_3$
O
1
2
3
$SeCH_3$
O
6
4
5
CN
-C=O
1+3
2
4
-CN
5
$-SeCH_3$
190
180
170
160
150
140
130
120
110
100
90
80
70
60
50
40
30
20
10
f1 (ppm)

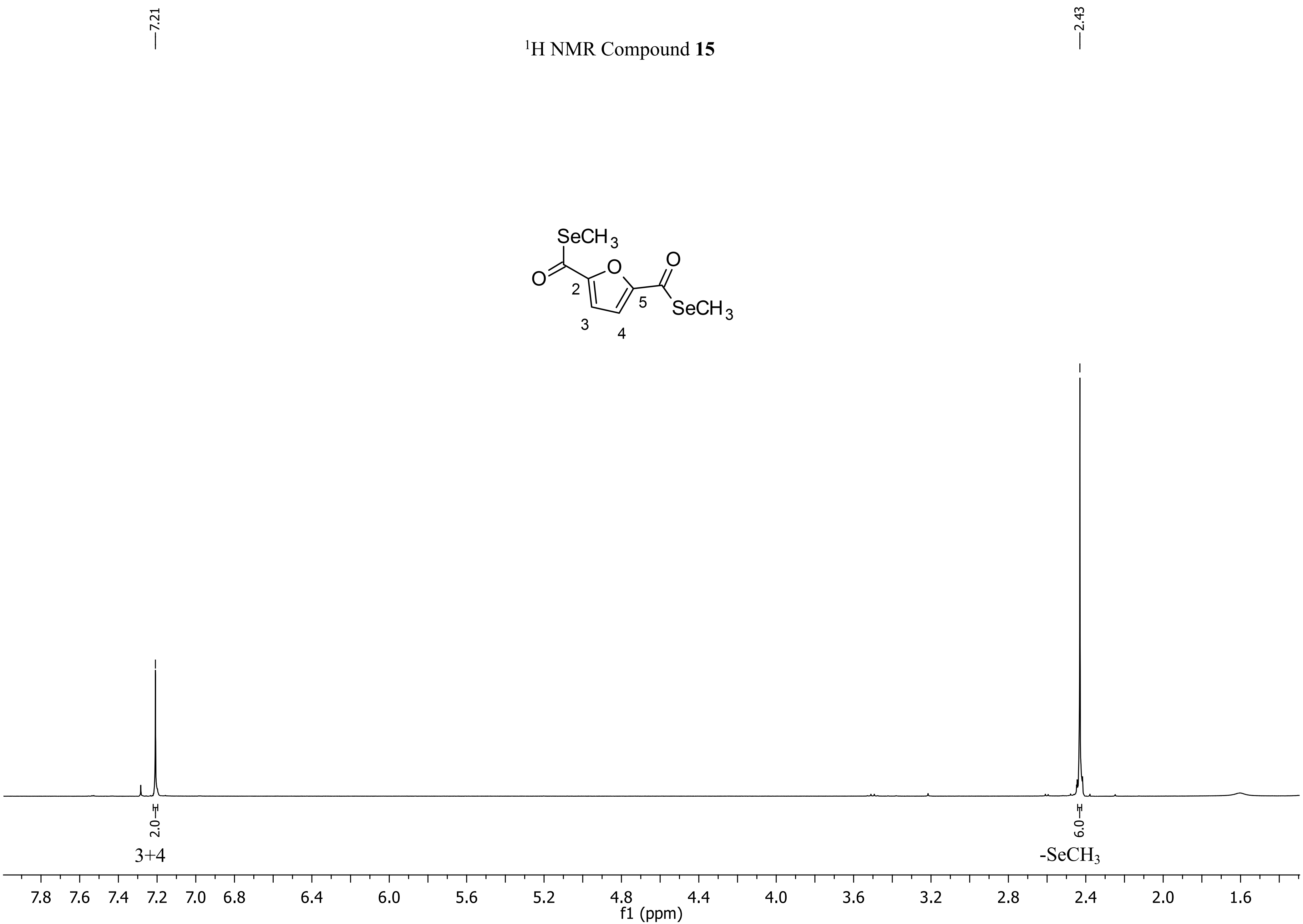
7.21
2.43
1H NMR Compound 15
SeCH3
O
O
O
2
5
SeCH3
3
4
2.0
6.0
3+4
-SeCH3
7.8 7.6 7.4 7.2 7.0 6.8 6.4 6.0 5.6 5.2 4.8 4.4 4.0 3.6 3.2 2.8 2.4 2.0 1.6
f1 (ppm)

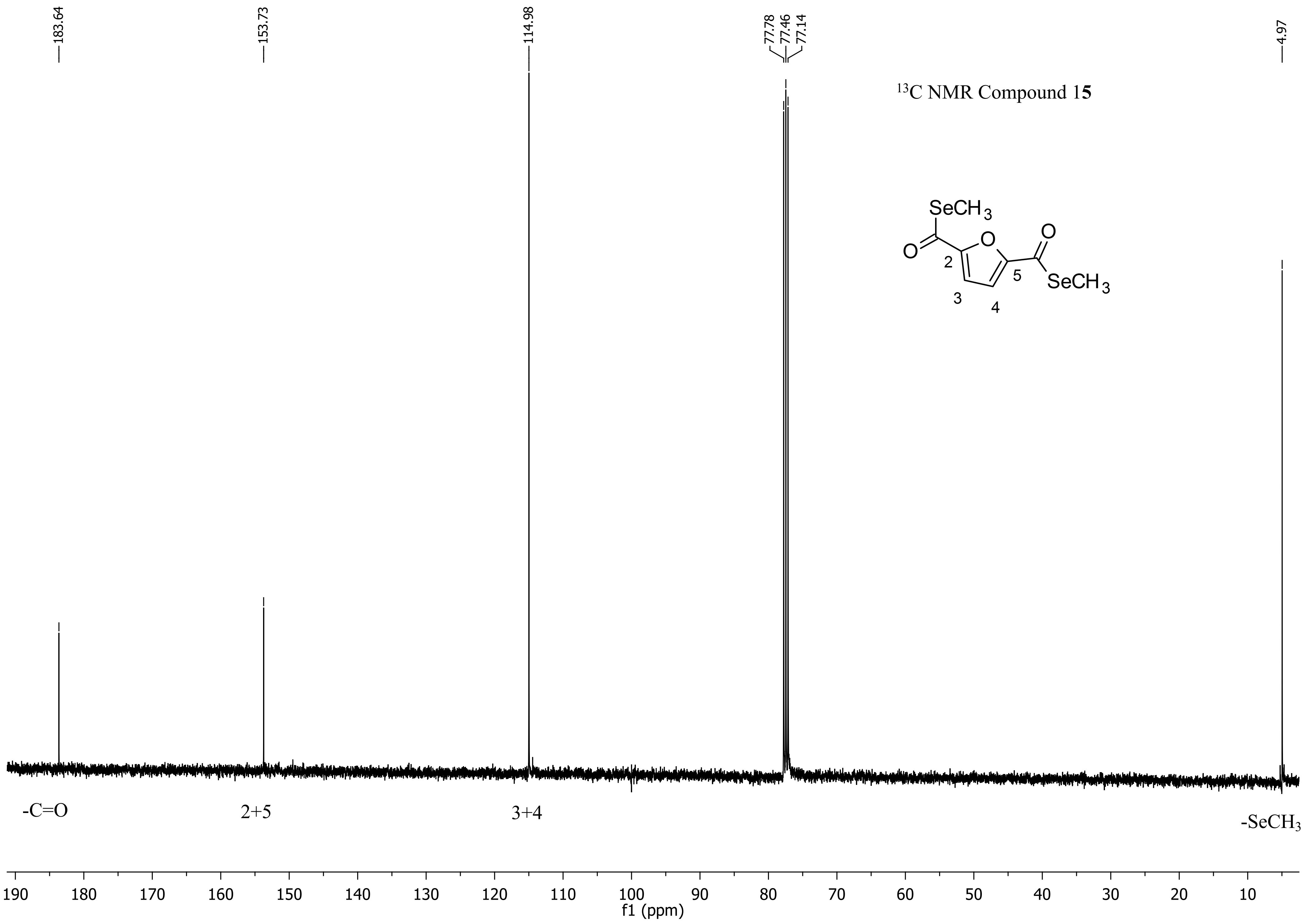

183.64
153.73
114.98
77.78
77.46
77.14
4.97
^{13}C NMR Compound 1**5**
SeCH3
O
O
O
2
5
SeCH3
3
4
-C=O
2+5
3+4
-SeCH3
190 180 170 160 150 140 130 120 110 100 90 80 70 60 50 40 30 20 10
f1 (ppm)
